# Structural and functional analysis of the three MIF4G domains of nonsense-mediated decay factor UPF2

**DOI:** 10.1093/nar/gkt1197

**Published:** 2013-11-23

**Authors:** Marcello Clerici, Aurélien Deniaud, Volker Boehm, Niels H. Gehring, Christiane Schaffitzel, Stephen Cusack

**Affiliations:** ^1^European Molecular Biology Laboratory, Grenoble Outstation, 6 rue Jules Horowitz, 38042 Grenoble Cedex 9, France, ^2^Unit of Virus Host-Cell Interactions, University of Grenoble Alpes-EMBL-CNRS, UMI 3265, 6 rue Jules Horowitz, 38042 Grenoble Cedex 9, France and ^3^University of Cologne, Institute for Genetics, Zuelpicher Street 47a, 50674 Cologne, Germany

## Abstract

Nonsense-mediated decay (NMD) is a eukaryotic quality control pathway, involving conserved proteins UPF1, UPF2 and UPF3b, which detects and degrades mRNAs with premature stop codons. Human UPF2 comprises three tandem MIF4G domains and a C-terminal UPF1 binding region. MIF4G-3 binds UPF3b, but the specific functions of MIF4G-1 and MIF4G-2 are unknown. Crystal structures show that both MIF4G-1 and MIF4G-2 contain N-terminal capping helices essential for stabilization of the 10-helix MIF4G core and that MIF4G-2 interacts with MIF4G-3, forming a rigid assembly. The UPF2/UPF3b/SMG1 complex is thought to activate the kinase SMG1 to phosphorylate UPF1 *in vivo*. We identify MIF4G-3 as the binding site and *in vitro* substrate of SMG1 kinase and show that a ternary UPF2 MIF4G-3/UPF3b/SMG1 complex can form *in vitro*. Whereas *in vivo* complementation assays show that MIF4G-1 and MIF4G-2 are essential for NMD, tethering assays reveal that UPF2 truncated to only MIF4G-3 and the UPF1-binding region can still partially accomplish NMD. Thus UPF2 MIF4G-1 and MIF4G-2 appear to have a crucial scaffolding role, while MIF4G-3 is the key module required for triggering NMD.

## INTRODUCTION

Nonsense-mediated mRNA decay (NMD) is a eukaryotic mRNA quality control mechanism that recognizes transcripts with premature termination codons (PTC) and promotes their degradation. Thereby, NMD protects the cell from the potentially deleterious effects of C-terminally truncated proteins (1–6). In fact, ∼30% of all known disease-causing mutations in humans involve production of PTC-containing mRNAs (7). NMD also contributes to regulate the abundance of several physiological substrates, targeting 3–10% of the transcriptome in different organisms (8–10).

Nine NMD protein factors, SMG1-9, have been identified in most metazoans (11,12), and recently four additional factors (SMG10, RUVLB1, RUVBL2 and RPB5) have been added to the components of the NMD machinery (13). The three UPF (UP-Frameshift) proteins, UPF1 (SMG2), UPF2 (SMG3) and UPF3 (SMG4) constitute the conserved core of NMD and are found in almost all eukaryotes with a few possible exceptions among protists (14–16), suggesting an ancient evolutionary origin for NMD. UPF1 is an ATP-dependent RNA helicase that is directly involved in the recognition of terminating ribosomes stalled at a PTC (17–19). Human UPF2 (Q9HAU5) is an ∼140 kDa protein containing three conserved MIF4G (Middle portion of eIF4G) domains that are found in a number of proteins involved in RNA metabolism and translation such as the nuclear cap-binding protein CBP80 and eIF4G (eukaryotic initiation factor 4-gamma) (20). UPF2 interacts via its third MIF4G domain with UPF3b and via its C-terminal extremity with UPF1, thus forming the central component of the ternary complex of UPF proteins (21–23). UPF3b stably binds the exon junction complex (EJC) (24–26), which is deposited by the splicing machinery on the mRNA ∼24 nt upstream of the exon boundaries. The EJC functions as an enhancer of NMD efficiency in mammals (27–29) probably by serving as a recruitment platform for UPF3b (22,24,26) and UPF2 after mRNA export to the cytoplasm (16,30).

Ribosomes stalled at a PTC are recognized by the NMD factors UPF1 and SMG1 to form the transient SURF complex, which consists of SMG1-UPF1-eRF1/3 a, SMG8 and SMG9 (12,19). SMG1 is an ∼415-kDa serine/threonine-protein kinase, essential for NMD in human and *Caenorhabditis elegans* (31), that belongs to the phosphatidylinositol 3-kinase–related kinase (PIKK) protein family (32). The EM structure of SMG1 in complex with SMG9 has been reported at 24 Å showing a characteristic head and arm architecture as observed previously for DNA-PKcs, another member of the PIKK family (33). Interaction of the SURF complex with NMD factors UPF2 and UPF3b positioned on a 3′ EJC is suggested to activate SMG1 to phosphorylate UPF1 in the ‘decay-inducing complex’ (DECID). UPF1 phosphorylation by SMG1 in the DECID leads to translation termination and dissociation of eRF3a from UPF1 (12,19). In addition, NMD factors SMG5-7 are recruited, which promote mRNA decay (34,35) and ultimately dephosphorylation of UPF1 (36). A major role in the interaction between the SURF and UPF2/3b-EJC complexes is played by UPF2 binding to UPF1. This interaction is mediated by the intrinsically disordered C-terminal extremity of UPF2, which structures on binding to the CH-rich domain of UPF1 (15). Moreover, an interaction between UPF2 and the SMG1 C-terminal region containing the kinase domain has been reported (19).

**Figure 1.**
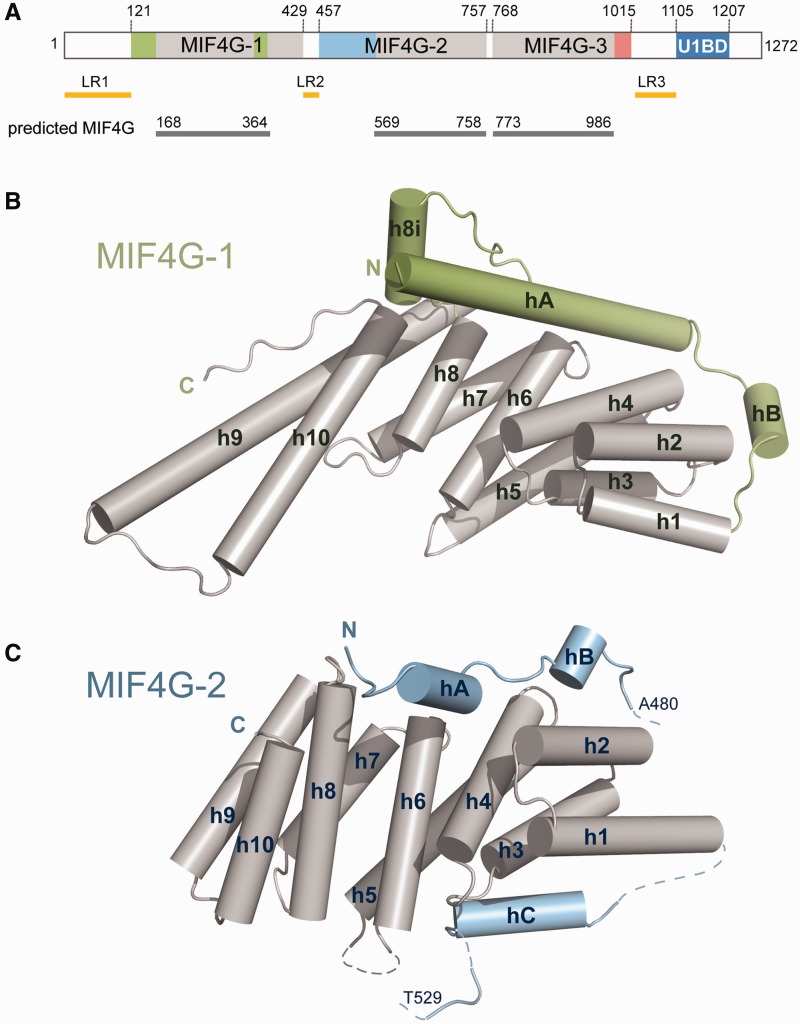
Crystal structures of UPF2 MIF4G-1 and MIF4G-2 domains. (**A**) Schematic representation of UPF2 indicating the domain boundaries (gray) of the conserved MIF4G domains according to SMART prediction. Colors (green, blue and pink) mark regions of the MIF4G domains that are not part of the classical 10-helix MIF4G domain fold. U1BD indicates the UPF1 binding domain. The N-terminal acidic low complexity region (residues 1–120) is indicated as LR1 (Linker Region 1). The disordered loop connecting MIF4G-1 and MIF4G-2 (residues 430–457) is indicated as LR2. The C-terminal acidic low complexity region (residues 1016–1104) connecting MIF4G-3 with U1BD is indicated as LR3 (**B**) Structure of MIF4G-1. The 10 helices (gray) of the classical MIF4G domain fold are numbered from 1 to 10. The N-terminal extension of the domain that folds into two helices (hA and hB) and the additional helix (h8i) inserted between h8 and h9 are depicted in green. The elongated helices h9 and h10 form a long coiled coil projecting from the otherwise compact domain. (**C**) Structure of MIF4G-2. The helix annotation is as in B with the N-terminal extension depicted in blue. It folds into three helices (hA, hB and hC), which are connected by partially disordered loops (shown as dashed lines). The long disordered loop between residues Ala480 and Thr529 is not represented.

A recent cryo-EM structure of the UPF1/2/3-EJC complex indicates that UPF2 acts as a central scaffolding protein with a ring-like arrangement of the MIF4G domains (37). According to the quasi-atomic model, UPF2 forms the crucial contacts with UPF1, UPF3b and the EJC and positions UPF1 toward the 3′-end of the mRNA where it could exert its helicase activity during mRNA degradation (37). High-resolution structural information of UPF2 is limited to the structure of the MIF4G-3 domain in complex with the RNP domain of UPF3b (16) and the UPF1-binding region in complex with UPF1 (15). The latter is separated from MIF4G-3 by a conserved low-complexity acidic region (88 residues, ∼50% Asp/Glu, indicated as LR3 in Figure 1A), which is likely to be disordered (15,23,38). Neither the structure nor the particular roles of the first two MIF4G domains of UPF2 are known. MIF4G domains have a conserved 3D structure, comprising an elongated bundle of antiparallel helices, despite high sequence divergence. They are thought to serve as molecular platforms for the recruitment of interaction partners (20,39–42) but could also be molecular spacers or scaffolds to correctly position distant functional regions.

Here, we present the atomic resolution crystal structures of the N-terminal (MIF4G-1) and middle (MIF4G-2) domains of UPF2 and a lower resolution crystal structure of the combined UPF2 MIF4G-2/MIF4G-3 domains. These structures allow a refinement of the quasi-atomic model of the entire UPF-EJC complex. Furthermore, we test the importance of UPF2 MIF4G-1 and MIF4G-2 domains for NMD *in vivo* and identify the UPF2 MIF4G-3 domain as the SMG1 interaction site. We also show that SMG1 kinase phosphorylates UPF2 MIF4G-3 domain *in vitro,* mainly at the serine residue 1046. *In vivo*, however, the UPF2 mutation S1046A did not interfere with NMD in complementation experiments. These results highlight the central role of the UPF2 MIF4G-3 domain in the DECID complex since it interacts with both UPF3 and SMG1 kinase, perhaps activating the latter to phosphorylate UPF1.

## MATERIALS AND METHODS

Full methods are given in the Supplementary Material.

### Crystallographic data collection and structure determination

Crystallographic statistics are given in Supplementary Table S1. All data collection was performed at 100 K at the European Synchrotron Radiation Facility. The data were integrated with XDS (43) and analyzed with CCP4i. Molecular replacement was performed with PHASER (44), model building with COOT (45) and refinement with REFMAC5 (46,47). The structures of both MIF4G-1 and MIF4G-2 were solved *de novo* by selenomethionine (SeMet) Single Anomalous Dispersion (SAD), using SHELXD (48) to find sites and SHARP (49) for refinement and phasing. For the low-resolution combined MIF4G-2/MIF4G-3 domain structure, molecular replacement using PHASER (44) using the individual domains, gave an unambiguous unique solution with log likelihood gain of 220. This was confirmed by correspondence of predicted methionine positions with anomalous difference peaks using data from MIF4G-2/MIF4G-3 crystals grown with SeMet. Due to the low resolution, no further refinement was performed.

### Generation of the quasi-atomic UPF-EJC model

Starting from the EM reconstruction of the UPF-EJC complex (EMD-2048) and the corresponding quasi-atomic model [Melero *et al.* (37)], we replaced the UPF2 MIF4G domains and the UPF3b RNA-recognition motif (RRM) domain by our crystal structure of MIF4G-1 domain and by the MIG4G-2/3/UPF3b RRM complex. We used Chimera (50) to obtain the best correlation coefficient for the placement of the domains into the density. To avoid a clash with MIF4G-1 and MIF4G-2 (both are larger than the MIF4G homology model used in the original atomic model), the EJC had to be moved by 14 Å away from UPF2. Similarly, the UPF1 CH-domain was moved by 5 Å away from UPF2 MIF4G-3 (Supplementary Figure S7). The figures were generated with PyMOL (DeLano Scientific). In the rendering of the cryo-EM map, the density cutoff was set for the display of the envelope to represent ∼130% of the *a priori* estimated volume.

### *In vivo* NMD assays

UPF2 tethering assay with the β-globin 4MS2 construct and the transfection control (wt300+e3) were described previously (24,51). For NMD rescue experiments, HeLa cells were transiently transfected with 100 pmol siRNA targeting UPF2 or luciferase. On the next day, cells were split 1:2 into 10-cm plates and 24 h later transfected with 200 pmol siRNA. The next day, cells were transfected by calcium phosphate precipitation with GFP and siRNA resistant UPF2 expression plasmids and with the plasmid encoding the reporter mRNA (TPI-HBB) (52).

### Surface plasmon resonance

Surface plasmon resonance (SPR) experiments were performed on a BIAcore 3000 using SA sensor chips (GE-Healthcare). One flow cell was not functionalized to be used as a background control. The second flow cell was functionalized with purified SMG1 to a density of ∼2000 RU. UPF2/UPF3b solutions were injected at 25 µl/min during 3 or 4 min followed by a 10-min dissociation phase. The surfaces were regenerated by 1-min injection of 0.5 M and 1 M NaCl solutions. The data were analyzed with the Biacore evaluation software by subtracting both the control flow cell and the buffer injection curve. Apparent equilibrium dissociation constant (K_D-app_) were determined using the RU values measured 10 s before the end of the association phase for all curves (RU_max_). These RU_max_ were plotted as a function of protein concentration and fitted assuming a one binding site model.

### Data deposition

Coordinates and structure factors for the UPF2 MIF4G-1 and MIF4G-2 domains are deposited in the Protein Data Bank with accession codes 4CEM and 4CEK, respectively.

## RESULTS

For structural studies of human UPF2 we initially used a construct comprising residues 121–1031 that encompasses all three predicted MIF4G domains but excludes the N-terminal low-complexity region (indicated as LR1 in Figure 1A) and the C-terminal acidic region as well as UPF1 binding domain (U1BD) (Figure 1A). UPF2(121–1031) was expressed in *Escherichia coli*, purified and subjected to crystallization trials, but no hits were obtained. Limited proteolysis of UPF2 (121–1031) led to the identification of three proteolytically stable fragments (one of ∼30 kDa and two of ∼45 kDa), each starting from residue 121 as determined by N-terminal sequencing (Supplementary Figure S1A). This information combined with sequence conservation and secondary structure [NPS@, (53)] and disorder [DisEMBL, (54)] predictions provided the basis for the design of different UPF2 constructs comprising a single MIF4G domain or two domains in tandem. Three well-behaved constructs were successfully crystallized: (i) residues 121–486 encompassing the MIF4G-1 domain, (ii) residues 455–757 encompassing the MIF4G-2 domain and (iii) the combined MIF4G-2/MIF4G-3 domains (residues 455–1054) (Supplementary Figure S1B). We solved the structure of UPF2 MIF4G-1 and MIF4G-2 domains at 2.6 and 2.4 Å resolution, respectively (Figure 1B and C), using SAD on seleno-methionine substituted protein crystals. Subsequently, the structure of the combined MIF4G-2/MIF4G-3 domains was determined at 5.4 Å resolution using molecular replacement. Crystallographic data are summarized in Supplementary Table S1.

### UPF2 MIF4G-1 domain has an extended coiled coil and two capping helices

UPF2 MIF4G-1 contains the canonical 10-helix core MIF4G fold (residues 168–429) with five pairs of antiparallel α-helices forming an N-terminal four-helix bundle (helices h1–h4) and two parallel layers composed of three helices each (helices h5–h10) (Figure 1B and structure annotated sequence alignment in Supplementary Figure S2). In addition, the domain displays three interesting features: (i) helices h9 and h10 are highly elongated at their C- and N-terminus, respectively, to form a long coiled coil that protrudes away from the rest of the domain into the solvent, the loop at the extremity being poorly ordered but still possible to be modeled (Figure 1B, Supplementary Figure S3A); (ii) an additional helix (h8i) is inserted between helices h8 and h9 via two extended loops (residues 324–344) (Figure 1B, Supplementary Figure S3B); (iii) the core MIF4G fold is preceded at the N-terminus by an extended α-helix (hA, residues 121–149) followed by a loop and a second shorter α-helix (hB) connecting to helix h1 (residues 150–167) (Figure 1B); the N-terminus of helix hA is packed against helix h8i (Figure 1B and Supplementary Figure S3B and C).

**Figure 2.**
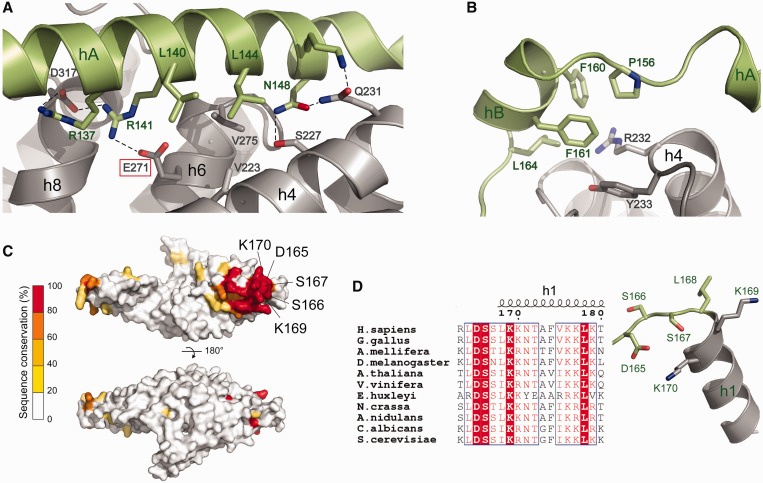
Interaction of the capping helices and adjacent loops with the MIF4G-1 core domain. Helix annotation and color code as in Figure 1B. (**A**) Helix hA interacts tightly with helices h4, h6 and h8 of the core domain. Amino acids involved in the interaction are shown in stick representation. The interaction involves hydrophobic interactions, hydrogen bonds and salt bridges. Notably, glutamate (E271) of the conserved FIGEL signature motif of MIF4G domains in helix h6 interacts with arginine 141 of helix hA. (**B**) Helix hB and its N-terminal loop interact via hydrophobic residues with the C-terminal tip of helix h4 of the conserved MIF4G fold. (**C**) Mapping of phylogenetically conserved residues on the solvent-accessible surface of MIF4G-1 based on the sequence alignment displayed in Supplementary Figure S2. Highly conserved residues (depicted in red) form a patch around the N-terminus of helix h1. For clarity a cartoon representation of MIF4G-1 in the same orientation is given in Supplementary Figure S3C. (**D**) Sequence alignment of the highly conserved 164-LDSSLKKNT-172 motif of UPF2 from representative species from yeast to human (left panel); 3D organization of residues 165–170 adjacent to helix h1 at its N-terminus (right panel).

MIF4G-1 helices hA, hB as well as their adjacent loops make extensive contacts with the 10-helix MIF4G core domain, in particular with helices h4, h6 and h8 (Figure 2A and B). This packing involves both hydrophobic and charged interactions. Notably, Arg137 and Arg141 on hA form hydrogen bonds and salt bridges with Asp317 and Glu271 on the core MIF4G domain, and Leu144 on hA interacts with Val223 and Val275 on h4 and h6, respectively (Figure 2A). Pro156, Phe160, Phe161 and Leu164 on hB form a hydrophobic patch interacting with Arg232 and Tyr233 on helix h4 (Figure 2B). Helix h8i and its adjacent loops pack against the MIF4G domain as well, establishing extensive hydrophobic and charged interactions with helices h6, h7, h8 and h9 (Supplementary Figure S3B). Helix h8i stabilizes the N-terminus of helix hA with mainly hydrophobic interactions (Supplementary Figure S3B). Overall, helices hA, hB and h8i create an extensive network of interactions with the 10 conserved helices of MIF4G-1 core MIF4G domain.

A highly conserved motif, 164-LDSSLKKNT-172 (Supplementary Figure S2), is located on the first turn of h1 and the loop immediately preceding it (Figure 2C and D). Mapping of phylogenetically conserved residues on the solvent-accessible surface of MIF4G-1 shows that the motif and a series of downstream conserved charged residues (Lys176, Lys177, Lys179, Asp192, Lys200, Glu204 and Glu211, Supplementary Figure S2) define a conserved patch at one extremity of the domain (Figure 2C). Asp165 and Lys170 side chains establish a salt bridge, while Ser167 N-terminally caps h1 by forming a hydrogen bond with the backbone amino group of Lys170, both contributing to fix the 3D configuration of the motif. Absolutely conserved Ser166, on the other hand, is solvent exposed and does not interact with other residues. Phosphorylation of one or both of the serines in this conserved motif has been reported to be required for NMD in yeast [(55), see discussion].

Residues 430–486 at the C-terminus of the MIF4G-1 crystallization construct are disordered in the crystal structure. This is consistent with the fact that the folded part of MIF4G-2 starts at residue 458 (see below), the region 430–457 being a flexible connecting loop between MIF4G-1 and MIF4G-2 (indicated as LR2 in Figure 1A) that is predicted to be disordered by DisEMBL and NPS@.

### N-terminal capping helices also occur in the UPF2 MIF4G-2 domain

**Figure 3.**
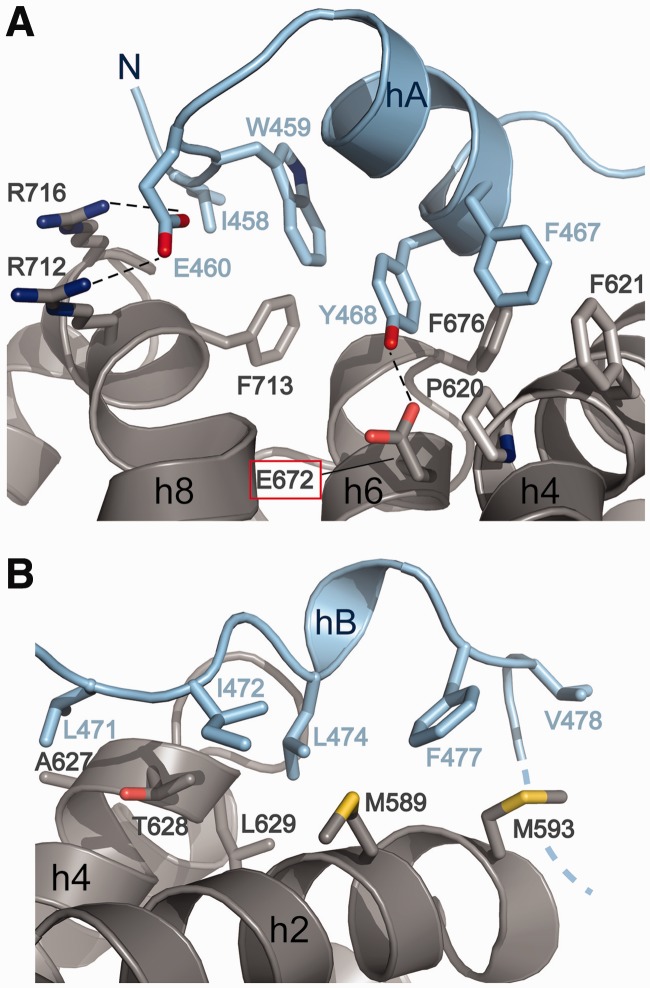
Interaction of the capping helices and adjacent loops with the MIF4G-2 core domain. Helix annotation and color code as in Figure 1C. (**A**) Helix hA and its N-terminal loop interact with helices h4, h6 and h8 of the core domain. Amino acids involved in the interaction are shown in stick representation. The interaction involves hydrophobic interactions, hydrogen bonds and salt bridges. Notably, glutamate (E672) of the conserved FIGEL signature motif in helix h6 interacts with tyrosine 468 of helix hA. (**B**) Helix hB and its adjacent loops interact via hydrophobic residues with helix h2 and h4 of the conserved MIF4G core.

The crystal structure of UPF2 MIF4G-2 (residues 455–757) shows that the core MIF4G fold of 10 antiparallel helices (residues 561–756) (Figure 1C) is preceded by an ∼100 amino acid region (residues 458–560) that contains additional elements essential for stability. The first part of this N-terminal region (458–478) folds into two short α-helices (hA and hB), which together with the connecting loops pack against helices h2, h4, h6 and h8 (Figure 3A and B). The interaction is mainly hydrophobic, involving Ile458, Trp459, Phe467 and Tyr468 of helix hA and Pro620, Phe621, Phe676 and Phe713 of h4, h6 and h8 (Figure 3A). Two salt bridges are also established between Glu460 and Arg712 and Arg716 on helix h8 (Figure 3A). Helix hB and its adjacent loops pack against helices h2 and h4 via hydrophobic interactions (Figure 3B). Residues 481–558 following helix hB are largely disordered in the crystal structure (apart from the short helix hC) and indeed this region is highly variable between species (Supplementary Figure S2). Similar to MIF4G-1, the N-terminal region stabilizes the interhelical packing of the MIF4G-2 domain helices. In fact, residues 458–478 are evolutionarily conserved within UPF2 (Supplementary Figure S2), and the expression of MIF4G-2/3 constructs lacking this capping region yields mostly insoluble protein (Supplementary Figure S3D). Notably, superposition of MIF4G-1 and MIF4G-2 shows that the capping helices hA and hB in the two domains pack against the same region of helices h2, h4, h6 and h8 (Supplementary Figure S4A).

### Interaction of MIF4G-2 with MIF4G-3

**Figure 4.**
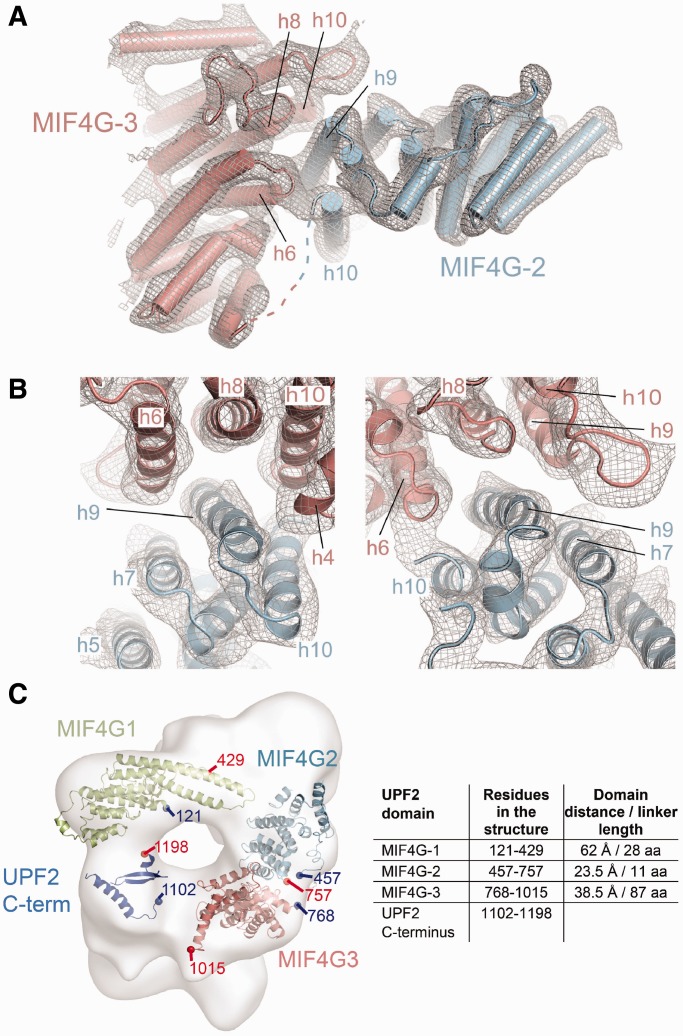
Crystal structure of combined MIF4G-2 and MIF4G-3 domains of UPF2. (**A**) Overview showing the perpendicular orientation of the two interacting domains with MIF4G-2 in blue and MIF4G-3 in pale pink. The 2mFo-DF electron density obtained by molecular replacement (at 5.4 Å resolution and contoured at 1σ) within 3 Å from the MIF4G-2 and MIF4G-3 domains coordinates is depicted as a gray mesh. The dotted line represents the 10 residues linking h10 of MIF4G-2 to h1 of MIF4G-3. (**B**) Close-up of the interacting helices. Helix h9 of MIF4G-2 inserts into a cavity formed by helices h6, h8 and h10 of MIF4G-3. The N-terminal part of MIF4G-2 h9 interacts also with h4 of MIF4G-3 (left panel). Helix h10 of MIF4G-2 contacts h6 of MIF4G-3 as evidenced by the connecting electron density between the two helices (right panel). (**C**) Refined quasi-atomic model of human UPF2 using the UPF2 C-terminus [Clerici *et al.* (15)], UPF2 MIF4G-1 (this study) and MIF4G-2/MIF4G-3 (this study) crystal structures into the cryo-EM reconstruction of the UPF1/2/3-EJC complex [EMD-2048, Melero *et al.* (37)]. The table summarizes the UPF2 domain boundaries, the distances between the UPF2 domains in the quasi-atomic model as well as the length of the linker peptides that connect these domains with the subsequent domain.

We solved the structure of the combined MIF4G-2/MIF4G-3 domains by molecular replacement at 5.4 Å resolution, using the two separate domains as search models (Figure 4A). To validate this structure we also collected anomalous scattering data on seleno-methionine-substituted MIF4G-2/MIF4G-3 crystals. The position of the methionine residues in the molecular replacement two domain model coincided with the selenium atoms revealed in the anomalous difference map, confirming the placement of the MIF4G-2 and MIF4G-3 domains (Supplementary Figure S5). The structure of the combined MIF4G-2/MIF4G-3 domains reveals a rigid assembly with the two domains being orthogonally oriented with respect to each other (Figure 4A). The buried surface area is estimated to be ∼750 and ∼639 Å^2^ for MIF4G-2 and MIF4G-3, respectively. The domains are connected via a short 11-amino acid-long linker (residues 757–767), which stretches the 23 Å distance between the C-terminus of MIF4G domain 2 and the N-terminus of MIF4G domain 3. The interaction is mediated mostly by MIF4G-2 helix h9, which inserts in a concave surface created by MIF4G-3 helices h6, h8 and h10 (Figure 4B).

The N-terminal part of MIF4G-2 helix h9 contacts the loop between MIF4G-3 helices h9 and h10 as well as the N-terminal part of helix h10. The C-terminal part of MIF4G-2 helix h9 interacts with the N-terminus of MIF4G-3 helix h4 (Figure 4B). An additional interaction is visible between the C-terminus of MIF4G-2 helix h10 and the N-terminus of MIF4G-3 h6.

The MIF4G-3 surface involved in the contact with MIF4G-2 is located opposite to the UPF3 binding site and therefore does not interfere with the UPF2-UPF3 interaction (Supplementary Figure S6). Superposition of MIF4G-3 in the UPF2/UPF3b complex (16) and in the MIF4G-2/MIF4G-3 structure indicates that the UPF3 RRM does not contact the UPF2 MIF4G-2 domain (Supplementary Figure S6).

### UPF2 MIF4G domain arrangement in the context of the UPF-EJC complex

Recently, a cryo-EM structure of the UPF1/2/3-EJC complex has been determined and used to derive a quasi-atomic model of the entire complex using previously known crystal structures of UPF1 bound to UPF2-U1BD, UPF2 MIF4G-3 bound to UPF3b RRM and the RNA-bound EJC (37). The UPF2 MIF4G-1 and MIF4G-2 domains were modeled only as core MIF4G domains. The results suggest that the MIF4G domains of UPF2 adopt, together with the UPF1 zinc-knuckle (CH) domain, a ring-like structure, which forms the central scaffold for complex assembly (37). We placed the crystal structure of MIF4G-1 and the MIF4G-2/3 assembly into the EM density of the UPF-EJC complex (EMD-2048) using Chimera (50) (Figure 4C, Supplementary Figure S7). The updated quasi-atomic model indicates that (i) the long coiled coil formed by helices h9 and h10 protruding from MIF4G-1 is in close proximity to and could contact the EJC, (ii) the loop between h9 and h10 contacts MIF4G-2 and (iii) the MIF4G-2 domain is positioned close to the Y14 subunit of the EJC likely forming a contact (Supplementary Figure S7). In conclusion, these observations support the proposed scaffolding function of UPF2 MIF4G domains (37) and indicate that UPF2 MIF4G-1 and MIF4G-2 domains are responsible for the positioning of the EJC in the UPF-EJC complex.

### Role of MIF4G domains 1 and 2 on NMD *in vivo*

**Figure 5.**
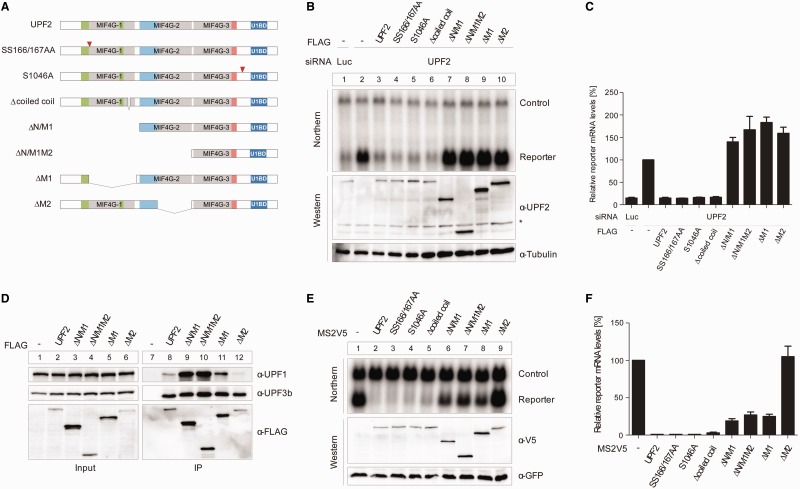
*In vivo* NMD tethering assays. UPF2 MIF4G-1 and -2 are required for NMD *in vivo.* (**A**) Schematic overview of UPF2 constructs used in the NMD analysis. UPF2 is represented as in Figure 1A; SS166/167AA and S1046A point mutant positions are indicated by red triangles. In the coiled coil deletion construct residues 370–406 were replaced by an Ala-Gly-Gly-Gly-Ala linker. (**B**) UPF2 complementation assay. The indicated siRNAs (Luc or UPF2) were transfected into HeLa cells. Next, plasmids encoding N-terminally FLAG-tagged UPF2 variants, the TPI-HBB PTC48 reporter mRNA and the LacZ-HBB control mRNA were transfected. Total RNA was analyzed by northern blotting. Protein samples were analyzed by western blotting using FLAG and tubulin (loading control) antibodies. The asterisk represents a nonspecific band. (**C**) Quantification of the relative reporter mRNA levels shown in the UPF2 complementation assay in (B), normalized to the control mRNA levels. (**D**) UPF1-UPF2-UPF3 co-immunoprecipitation assay. HeLa cells were transfected with the indicated FLAG-tagged expression plasmids. Immunoprecipitation was performed using FLAG beads and co-precipitated UPF1 and UPF3b were detected by western blotting using specific antibodies. (**E**) UPF2 tethering assay. HeLa cells were transfected with plasmids encoding the indicated N-terminally MS2V5-tagged UPF2 variants and co-transfected with plasmids for the reporter (β-globin 4MS2) and control (wt300+e3). Total RNA was isolated and analyzed by northern blotting. Expression levels of the tethered proteins were visualized by western blotting using a V5 antibody. GFP served as transfection control. (**F**) Quantification of the reporter mRNA levels shown in the UPF2 tethering assay in (E), normalized to the expression levels of the control mRNA.

To evaluate the role of MIF4G-1 and MIF4G-2 in NMD we performed a UPF2 complementation assay in HeLa cells. In this assay the abundance of a NMD-competent triosephosphate isomerase (TPI) reporter mRNA harboring a PTC at position 48 is measured on siRNA silencing of endogenous UPF2 and rescue by transfection with different siRNA insensitive UPF2 expression constructs. Four UPF2 MIF4G-1 and/or MIF4G-2 deletion mutants were generated (Figure 5A): UPF2 ΔM1 and UPF2 ΔM2, lacking the core MIF4G-1 domain (residues 168–431) and the core MIF4G-2 domain (residues 569–758), respectively; UPF2 ΔN/M1 lacking MIF4G-1 and the preceding N-terminal region (residues 1–455) and UPF2 ΔN/M1M2 lacking MIF4G-1, MIF4G-2 and the preceding N-terminal region (residues 1–757). All four constructs are impaired in their ability to support NMD (Figure 5B lane 7–10 and Figure 5C) compared with wild-type UPF2 (Figure 5B lane 3 and Figure 5C). We noticed that these mutants show enhanced expression levels compared with wild-type UPF2 (Figure 5B), suggesting that their inability to support NMD is not the result of decreased protein stability. We also excluded that this effect is due to altered cellular localization since all the tested mutants localize in the cytoplasm as wild-type UPF2 (Supplementary Figure S8A). Next, we verified that the UPF2 mutants retained the ability to co-immunoprecipitate UPF1 and UPF3 (Figure 5D). Taking into account the differences in expression levels, UPF2 ΔM1 and ΔM2 show a virtually unaltered ability to interact with UPF1, whereas UPF2 ΔN/M1 and ΔN/M1M2 show enhanced co-immunoprecipitation of UPF1 (Figure 5D). UPF3 binding is unaltered in all four mutants. Altogether, these results demonstrate the essential role of MIF4G-1 and MIF4G-2 during NMD for reasons independent of protein stability, cellular localization or UPF3 and UPF1 binding. We also noticed that in the presence of these UPF2 constructs the reporter mRNA levels are higher than in the absence of transfected UPF2 (Figure 5B, lane 2 and Figure 5C). This dominant negative effect is likely due to the UPF2 mutants sequestering endogenous UPF1 and UPF3 and thus further reducing NMD in addition to endogenous UPF2 silencing.

We used the same UPF2 deletion constructs in a NMD tethering assay (24,51). In this assay, UPF2 is directly tethered to a beta-globin mRNA downstream of the PTC, thus bypassing the need for the interaction of UPF2 with UPF3-EJC for its recruitment to the mRNA. In this assay UPF2 ΔM1, ΔN/M1 and ΔN/M1M2 are able to support NMD with a partial impairment (Figure 5E lane 6–8 and Figure 5F) compared with wild-type UPF2 (Figure 5E lane 2 and Figure 5F). This indicates that when UPF2 is directly tethered to the mRNA, MIF4G-1 and MIF4G-2 are partially dispensable for its downstream interaction with the SURF complex and the triggering of UPF1 phosphorylation. Surprisingly, the UPF2 ΔM2 construct appears to be completely inactive in the tethering assay (Figure 5E lane 9 and Figure 5F). This is unexpected since the lack of the MIF4G-2 domain in the UPF2 ΔN/M1M2 construct did not severely affect NMD. Thus, the mere absence of MIF4G-2 cannot explain the failure of UPF2 ΔM2 to support NMD. Rather, the unusual proximity, in this construct, of the MIF4G-1 domain to the MIF4G-3 and UPF1 binding domains, may sterically interfere with UPF1 and SMG1 binding, thus affecting NMD.

The fitting of the UPF2 MIF4G domains in the EJC-UPF envelope (Supplementary Figure S7) suggests that the MIF4G-1 coiled coil contacts the EJC, thus possibly having a key role in the stabilization of the EJC-UPF complex. To test this possibility, we designed a UPF2 construct (Δcoiled coil) where the protruding region of MIF4G-1 coiled coil (residues 370–406) is replaced by a short linker (Ala-Gly-Gly-Gly-Ala) (Figure 5A). Both in the NMD complementation (Figure 5B lane 6 and Figure 5C) and tethering assay (Figure 5E, lane 5 and Figure 5F), the loss of MIF4G-1 coiled coil does not interfere with NMD, suggesting that this is not the only region involved in stabilizing the EJC-UPF complex.

### SMG1 binds and phosphorylates UPF2 MIF4G-3

**Figure 6.**
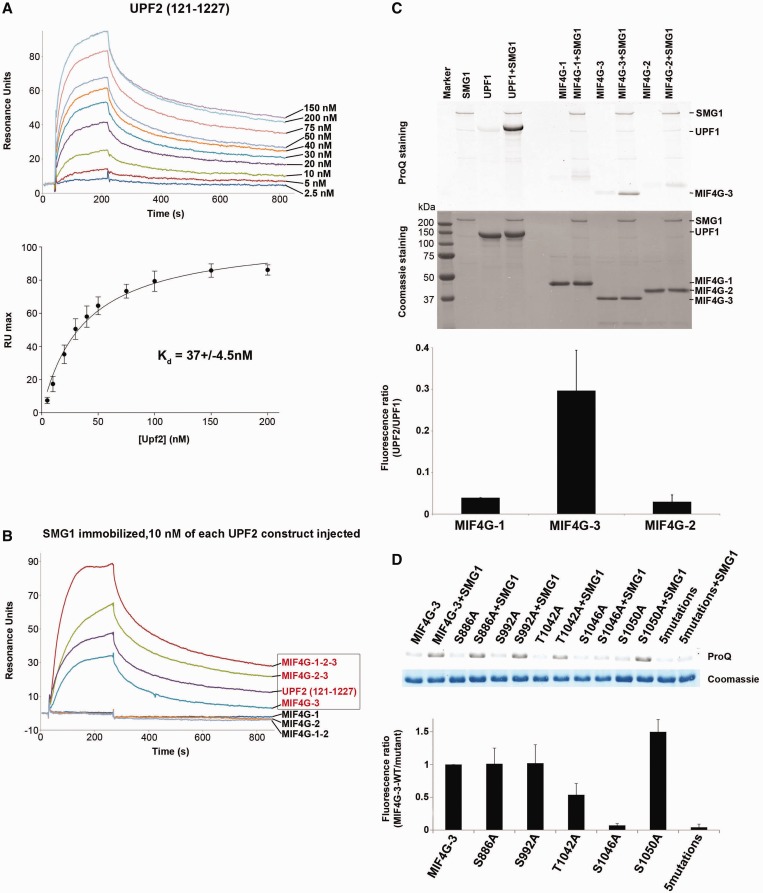
Characterization of the UPF2-SMG1 interaction. (**A**) Overlay of SPR sensograms of UPF2(121–1227) comprising all three MIF4G domains (above) interacting with immobilized SMG1. UPF2 (121–1227) was injected at concentrations ranging from 2.5 to 200 nM. The maximal resonance unit signal was plotted against the UPF2 concentration (below); the data points result from three independent experiments. The apparent dissociation constant K_D-app_ was determined assuming a 1:1 interaction of SMG1 and UPF2. (**B**) SPR interaction profile of different UPF2 constructs with immobilized SMG1. All UPF2 constructs were injected at a concentration of 10 nM. UPF2 constructs containing the MIF4G-3 domain are labeled with red text color. Noninteracting constructs containing MIF4G-1 and/or MIF4G-2 are labeled with black text color. (**C**) *In vitro* phosphorylation experiments of UPF2 MIF4G constructs with SMG1 kinase. SMG1 was mixed with different UPF2 MIF4G domain constructs and with UPF1 as a positive control. The reaction mixture was analyzed by SDS-PAGE and Pro-Q Diamond Staining (Life technologies) (top) staining only phosphorylated proteins. SMG1 is auto-phosphorylated and therefore stains in the experiment as well. The Coomassie-stained gel (middle) shows all proteins present the experiments. The phosphorylation signal after Pro-Q staining was quantified with a Typhoon scanner (bottom). Each bar presents the average of three independent experiments. (**D**) As for (**C**), SMG1 was mixed with different UPF2 MIF4G-3 mutants to assess *in vitro* phosphorylation. The reaction mixture was analyzed by SDS-PAGE and Pro-Q Diamond (top) and Coomassie staining (middle). The phosphorylation level of each mutant was quantified using the ratio of its fluorescence signal to the wild-type fluorescence signal (bottom). Each bar represents the average of four independent experiments.

Previous work suggested that the UPF1 kinase SMG1 also interacts with UPF2 (19). We therefore tested whether various truncated UPF2 constructs are able to interact with full-length recombinant SMG1 by SPR. Wild-type SMG1 was immobilized via an N-terminal SBP-tag onto a streptavidin sensor chip, and the UPF2 constructs were injected. First, we determined the apparent dissociation constant K_D-app_ using UPF2 containing all three MIF4G domains and the UPF1 binding domain (residues 121–1227). This measurement confirmed that SMG1 and UPF2 tightly interact with a K_D-app_ of 37 ± 5 nM (Figure 6A). Subsequently, we asked which UPF2 domain interacts with SMG1. To this end, we tested the individual MIF4G domains as well as UPF2 constructs carrying different combinations of MIF4G domains. We were able to demonstrate that MIF4G domain 3 is sufficient for SMG1 interaction. In contrast, MIF4G-1 and MIF4G-2 do not show binding to SMG1 (Figure 6B). Moreover, we measured for the MIF4G-3/SMG1 complex a K_D-app_ of 60 ± 5 nM (Supplementary Figure S9A), which constitutes a <2-fold reduction compared with the K_D-app_ of 37 ± 5 nM obtained for UPF2 (121–1227) and SMG1. These results strongly suggest that MIF4G-3 is the main determinant for UPF2-SMG1 complex formation.

Next, we investigated whether SMG1 is able to phosphorylate UPF2 MIF4G domains *in vitro*. We found that MIF4G-3, but not MIF4G-1 and MIF4G-2, is significantly phosphorylated by SMG1 in the presence of ATP (Figure 6C). MIF4G-3 shows about three times less phosphorylation as measured by Pro-Q staining compared with the known SMG1 substrate UPF1, which served as a positive control (Figure 6C). UPF1 has up to five Ser/Thr phosphorylation sites, which are recognized by SMG1 *in vivo* and *in vitro* (56). UPF2 (121–1227) carrying all three MIF4G domains is phosphorylated to a similar extent as MIF4G-3. We performed large-scale phosphorylation of UPF2 MIF4G-3, to purify the *in vitro* phosphorylated form. The pure domain was then subjected to mass spectrometry analysis. Three SMG1 phosphorylation sites were identified (Supplementary Table S2): Ser886, Ser992 and Thr1042, Ser1046 or Ser1050. Ser886 and Ser992 are located at the N-terminus of MIF4G-3 helices h7 and h11, respectively (Supplementary Figure S10). Residues Thr1042, Ser1046 and Ser1050 are located on the linker between the structured part of MIF4G-3 and the UPF2 C-terminus, which binds UPF1. In the UPF-EJC quasi-atomic model, Ser992, Thr1042, Ser1046 and Ser1050 are positioned at the free surface of the ring formed by UPF2 MIF4G domains (Supplementary Figure S10). Thus, they are likely to be exposed and accessible for SMG1 phosphorylation in the UPF-EJC complex. Next, each individual residue has been substituted to alanine and tested for *in vitro* phosphorylation by SMG1. All alanine mutants except Ser1046 are phosphorylated to a similar extent as the wild-type MIF4G-3 domain (Figure 6D). Moreover, mutation of all five Ser/Thr led to a similar phosphorylation signal as the single S1046A mutant (Figure 6D), indicating that Ser1046 is the main phosphosite for SMG1 in the UPF2 MIF4G-3 construct (residues 761–1054), which we used as substrate in our assays.

Next, we performed NMD complementation and tethering assays in HeLa cells to assess whether the UPF2 S1046A mutation would affect NMD *in vivo*. In both experiments, the S1046A mutation does not interfere with NMD *in vivo* (Figure 5B lane 5 and Figure 5E lane 4). To rule out the possibility that a single phosphorylation site mutation is not sufficient to produce a detectable effect on NMD efficiency, we tested a UPF2 construct carrying alanine mutations for all five identified phosphorylation sites and tested it in a complementation assay. Also for this construct we did not detect any effect on NMD *in vivo* (Supplementary Figure S8B). In addition, the serine residues 166 and 167, which are located in the highly conserved motif of MIF4G-1 and have been reported to be important for NMD in yeast (55), were both mutated to alanines. The mutation of these residues did not impair UPF2 in complementation as well as in tethering assays (Figure 5B lane 4 and Figure 5E lane 3).

### SMG1 and UPF3b can simultaneously bind to UPF2 MIF4G-3 domain

**Figure 7.**
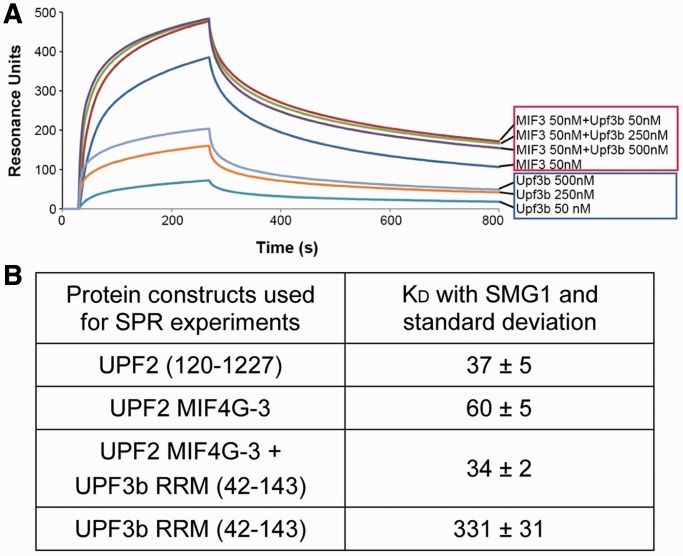
UPF2 MIF4G-3 binding to UPF3 and SMG1 is not mutually exclusive. (**A**) SPR interaction profile of UPF2 with SMG1 in the presence of increasing concentration of UPF3b RRM domain (red box), and profile of UPF3b RRM domain interacting with SMG1 (blue box). UPF2 was injected at a concentration of 50 nM in the presence of 0, 50, 250, 500 nM UPF3b. UPF3b alone was injected a concentrations ranging from 50 to 500 nM. (**B**) Apparent dissociation constants (K_D-app_) of the UPF protein constructs and SMG1 determined by SPR experiments, which identify the MIF4G-3 domain as the SMG1 binding domain and the existence of a ternary MIF4G-3/UPF3b/SMG1 complex.

Since UPF3b and SMG1 both bind to UPF2 MIF4G-3 domain, we tested whether this binding was mutually exclusive using two different approaches. First, we performed a competition experiment where the binding of MIF4G-3 at constant concentration (50 nM) to SMG1 was measured by SPR in the presence of UPF3b. Increasing concentrations of UPF3b (50, 250 and 500 nM) do not decrease the binding of UPF2/UPF3b to SMG1 (Figure 7A). This indicates that UPF3b and SMG1 do not compete for UPF2 binding and that the UPF2/UPF3b-SMG1 complex can form. In the case of overlapping binding sites, i.e. competition, addition of UPF3b to UPF2 would reduce the SPR signal. Rather, formation of the UPF3-MIF4G-3 complex leads to an increased signal in SPR (Figure 7A). Second, we measured by SPR the apparent dissociation constant between SMG1 and the preformed MIF4G-3/UPF3b complex. The apparent dissociation constant of SMG1 and UPF2 MIF4G-3 in complex with UPF3b (K_D-app_ = 34 ± 2 nM) and of SMG1 and MIF4G-3 alone (K_D-app_ = 60 ± 5 nM) is similar (Figure 7B and Supplementary Figure S11), confirming that the UPF3b RRM domain does not compete or interfere with the UPF2-SMG1 complex formation.

As a control, we tested the binding of UPF3b RRM domain (residues 42–143) to SMG1. Compared with UPF2, UPF3b shows an ∼10-fold weaker interaction with SMG1 (K_D-app_ = 331 ± 31 nM) (Figure 7B and Supplementary Figure S9B) with fast on and off rates. The UPF3b domain is highly charged at neutral pH (UPF3b isoelectric point is 8.8), and thus the observed binding could be due to nonspecific charged interactions.

## DISCUSSION

### UPF2 MIF4G domain 1 and 2 folds are stabilized by N-terminal capping helices

UPF2 is composed of three conserved tandem MIF4G domains and a C-terminal UPF1 interacting domain (Figure 1A). Structural information is available for the MIF4G-3 interacting with the RRM domain of UPF3b (16) and of the C-terminus of UPF2 in complex with UPF1 (15). Here, we present the crystal structures of the remaining UPF2 folded domains, the N-terminal MIF4G-1 and MIF4G-2 domains. In both cases, we found that the canonical 10-helical MIF4G fold is augmented by an N-terminal extension that packs onto the core and is essential for overall domain stability (Figure 1). The additional helices in the N-terminus, in particular helix hA, are located in a similar position in the MIF4G-1 and -2 domains and pack against helices h2, h4, h6 and h8 of the MIF4G core involving both hydrophobic and charged interactions (Figures 2 and 3). Consistent with the multiple interactions of the capping helices with the MIF4G core, deletion of the N-terminal extension renders the domains aggregation-prone, indicating that it has an important stabilizing function. We note that while UPF2 MIF4G-3 does not have an N-terminal extension (although its contact with MIF4G-2 may play a stabilizing role), it does have an extra C-terminal helix (h11) added to the core MIF4G fold. We provide a more extensive discussion of the structural conservation of MIF4G domains in the Supplementary Material.

### MIF4G-1 contains unique elongated helices

In addition to the N-terminal extension, MIF4G-1 contains two elongated helices (h9 and h10) (Figure 1). This is a striking and phylogenetically conserved feature of the UPF2 MIF4G-1 domain, although a similar extended coiled coil also occurs in the third, C-terminal, MIF4G-like domain of CBP80 but formed by helices equivalent to h8 and h9 and thus pointing in a different direction (40). When fitting the MIF4G-1 domain into the EM density of the UPF-EJC complex (37), we observe that the loop connecting h9 and h10 is in close proximity and likely to contact MIF4G-2 (Supplementary Figure S7). Thus, this protrusion may be important for stabilizing the correct 3D arrangement of the UPF2 MIF4G domains in the UPF-EJC complex, MIF4G-1 and MIF4G-2 otherwise being connected by a flexible linker. Moreover, MIF4G-1 h9 and h10 are in a position to establish contacts to the EJC in the complex (Supplementary Figure S7). Therefore, MIF4G-1 could contribute to the postulated scaffolding function of UPF2 (37) by positioning the EJC in the DECID complex. We tested this hypothesis with *in vivo* NMD assays (Figure 5) but found that deletion of the extended part of h9 and h10 does not affect NMD.

### MIF4G domain arrangement in UPF2

The crystal structure of the tandem MIF4G-2/MIF4G-3 domains shows that they form a rigid assembly with a substantial total buried surface area of ∼1400 Å^2^. The two domains are oriented perpendicular to each other with helices h9 and h10 of MIF4G-2 interacting with helices h4, h6, h8 and h10 of MIF4G-3 (Figure 4). Interestingly, in MIF4G-1 and 2, these helices (h4, h6, h8 and partly h10) are contacted by the N-terminal extension (hA, hB and adjacent loops). In contrast to the observed rigid juxtaposition of MIF4G-2 and MIF4G-3, limited proteolysis analysis of UPF2 (Supplementary Figure S1A) suggests that MIF4G-1 is flexibly linked with respect to MIF4G-2/MIF4G-3 in free UPF2 and only becomes fixed in position within the UPF-EJC complex.

Sequence analysis shows that proteins usually contain two or more MIF4G or MIF4G-like (HEAT or ARM) domains in tandem connected by predicted unstructured linkers (57,58). This is the case for CBP80, eIF4G and UPF2. The structure of CBP80 provides an example of three combined MIF4G/MIF4G-like domains (40) where the middle HEAT domain is the central core of the protein and the N-terminal MIF4G and the C-terminal HEAT domains pack against it, forming a compact assembly. CBP80 thus shows a globular shape different from the ring-like arrangement of UPF2 MIF4G domains, which is suggested by the UPF-EJC EM reconstruction (37). Consistently, the superposition of UPF2 MIF4G-2/MIF4G-3 to CBP80 MIF4G domains does not indicate any obvious similarity in the relative domain organization of the two proteins. Therefore, even though proteins containing tandem MIF4G/MIF4G-like domains may have a common ancestor [as suggested for eIF4G and CBP80 (58)], the structural arrangement of the MIF4G domains can clearly differ from protein to protein.

### UPF2 MIF4G-1 and -2 scaffold the UPF-EJC complex but have no direct function in interaction with the SURF complex

The EM structure of the UPF-EJC complex shows that UPF2 forms a ring-like scaffold for the assembly of the UPF-EJC complex (37). Our refined quasi-atomic model indicates that MIF4G-1 and 2 are key bridging factors between the EJC and the UPF1 CH domain and the UPF2 MIF4G-3 domain, respectively (Supplementary Figure S7).

By UPF2 complementation assays we tested the impact of UPF2 MIF4G-1 and MIF4G-2 deletion on NMD efficiency. All tested deletions completely impaired the ability of UPF2 to activate NMD of a reporter mRNA (Figure 5B and C), confirming the absolute requirement of MIF4G-1 and MIF4G-2 for NMD in cells. The same constructs were also used in a UPF2 tethering assays. In this situation it is likely that only the ability of the UPF2 variants to interact with the SURF complex and to activate UPF1 phosphorylation, which ultimately triggers mRNA degradation, is tested. Contrary to the complementation assays, UPF2 ΔM1, ΔN/M1 and ΔN/M1M2 were only partially impaired to support NMD in comparison with the wild type (Figure 5E and F). Thus, by the complementary use of these two assays we were able to uncouple MIF4G-1 and 2 functions in scaffolding the UPF-EJC complex (complementation) and in activating the downstream events (tethering). The diverging outcome of the two assays strongly indicates that MIF4G-1 and MIF4G-2 are strictly necessary for NMD to promote the correct assembly of the EJC and the UPF proteins, but are partially dispensable for the interaction of UPF2 with the SURF complex and triggering of UPF1 phosphorylation.

### MIF4G domain 3 plays a central role in the DECID complex

The critical ‘point-of-no-return’ in NMD is the activation of SMG1 kinase to phosphorylate UPF1 (31,59). This is achieved through association of the SURF complex with the UPF2/3-EJC complex to form the decay inducing complex (DECID) (19). Details of the UPF1 interaction with UPF2 have been revealed by radiographic crystallography (15) and with the UPF2/3-EJC complex by cryo-EM (37). Immunoprecipitation experiments indicate a direct interaction between UPF2 and the C-terminal region of SMG1 that contains the kinase domain (19). Here, we identify UPF2 MIF4G-3 as the interaction site with SMG1 kinase (Figure 6). We measured by SPR an apparent dissociation constant of 37 nM for UPF2 (121–1227) and SMG1 (Figure 6A), which is only slightly increased to 60 nM when the MIF4G-3 domain alone binds SMG1 (Supplementary Figures S9A and Figure 7B). No interaction between SMG1 and MIF4G domains 1 and 2 could be detected *in vitro* (Figure 6B). Our finding is further supported by the *in vivo* tethering assays that indicate that the deletion of the N-terminal region of UPF2 does not strongly interfere with UPF2 interaction with the SURF complex (Figure 5A).

The UPF2 MIF4G-3 domain is a relatively small domain that interacts stably with both MIF4G-2 (Figure 4) and the UPF3b RRM domain (16). Moreover, according to the UPF-EJC cryo-EM structure, UPF2 MIF4G-3 is also in close proximity with the UPF1 CH domain (37). Therefore, we investigated whether UPF2 MIF4G-3/UPF3b complex formation would interfere with UPF2 MIF4G-3/SMG1 interaction. The measured apparent dissociation constant of MIF4G-3 and SMG1 in the presence of UPF3b RRM domain (34 nM) is comparable with that of MIF4G-3 alone (60 nM), suggesting that UPF3b does not compete with UPF2 for SMG1 binding and that the trimeric UPF2/UPF3b/SMG1 complex can exist also *in vivo*.

SMG1 kinase is suggested to be downregulated by the SMG8/SMG9 heterodimer (12). SMG9 has been shown to bind to the N-terminal HEAT-repeat region of SMG1 and may act as a recruitment platform for SMG8, which, upon binding, induces conformational changes in SMG1 and inhibits its kinase activity (33). UPF2 has been reported to interact with the C-terminal region of SMG1 carrying the kinase domain (19), and therefore UPF2 likely does not compete with SMG8/SMG9 for binding the N-terminus of SMG1. Consequently, UPF2 could act directly on the kinase domain to activate SMG1.

### UPF2 is a SMG1 kinase substrate

Here we provide further evidence for a direct interaction of UPF2 with the SMG1 kinase domain. We observed that UPF2 MIF4G-3 is phosphorylated by SMG1 kinase *in vitro* (Figure 6C). A combination of mass spectrometry and *in vitro* mutational analyses identified three phosphosites: Ser886, S992 and Ser1046 but only the latter one is quantitatively phosphorylated *in vitro* (Figure 6D). Ser1046 is not part of a canonical SQ site. However, a Gln is present before the Ser1046 and this residue is located in a Glu-rich region. These two elements have been found in ATM/ATR phosphosites identified by mass spectrometry (60). According to the quasi-atomic model derived from the UPF-EJC complex reconstruction [Melero *et al.* (37)], Ser1046 is positioned on a flexible loop and thus likely to be exposed. Furthermore, it is positioned close to the UPF1 phosphosites and thus could be accessible by SMG1 (Supplementary Figure S10). Therefore, the identification of an *in vitro* phosphorylation site in MIF4G-3 confirms the direct interaction of UPF2 MIF4G-3 with the SMG1 kinase domain, possibly leading to activation of SMG1 to phosphorylate UPF1. However, mutation of Ser1046 or of all five identified phosphorylation sites are not sufficient to interfere with NMD in cells, neither in complementation assays nor in tethering assays (Figure 5). Consistent with our findings, human UPF2 has been reported to be a phosphoprotein (36), but it has not been identified as a SMG1 kinase substrate *in vivo*, nor has any functional role for human UPF2 phosphorylation been proposed. In contrast, UPF2 binding to SMG1 kinase is essential to trigger NMD (19).

In contrast, a study in *Saccharomyces cerevisiae* showed that yeast Upf2p is phosphorylated *in vivo* (55), most likely including serines 32 and/or 33 in the highly conserved motif near the beginning of the MIF4G-1 domain (30-LDSSIKRNT in yeast, see above and Figure 2C and D). Mutational analyses showed that the residues 21-DSS are important for interaction of Upf2p with the yeast-specific NMD factor Hrp1p and for triggering NMD *in vivo* (55). If this motif adopts the same configuration as observed in the human MIF4G-1 structure, only Ser32 (equivalent to Ser166 in human UPF2) is solvent exposed and could be phosphorylated. In fact, Ser32 is phylogenetically absolutely conserved. However, an interaction partner for this motif in higher eukaryote UPF2 remains to be identified. UPF2 constructs with mutations, S166A, S166D (data not shown) and SS166/167AA (Figure 5) in this motif were tested in tethering and complementation experiments. However, no effect on NMD was observed (Figure 5B and E), suggesting that the intact motif is not required for DECID complex formation. Therefore, it remains to be shown whether UPF2 phosphorylation by SMG1 (which has no homologue in yeast) or by any other kinase has a significant role in NMD in higher eukaryotes.

In summary, we elucidate here the structure of UPF2 MIF4G domains 1 and 2 and show that they exert a role in the structural organization of the EJC-UPF complex. UPF2 MIF4G domain 3 interacts with the SMG1 kinase domain and UPF3b and plays the key role in DECID complex formation and SMG1 activation, which triggers NMD.

## SUPPLEMENTARY DATA

Supplementary Data are available at NAR online.

## FUNDING

European Molecular Biology Laboratory EIPOD postdoctoral programme [fellowship to A.D.]; European Research Council [ERC Starting Grant 281331 ComplexNMD to C.S.]; Swiss National Science Foundation [Sinergia grant CRSII3_136254, Structure/Function analyses of PIKKs to C.S.]; International Graduate School in Development Health and Disease IGSDHD [fellowship to V.B.]; the Deutsche Forschungsgemeinschaft [SFB635, project B6 to N.H.G.]. Funding for open access charge: EMBL.

*Conflict of interest statement*. None declared.

## Supplementary Material

Supplementary Data
