# Supplementary material for: Structural and functional analysis of the three MIF4G domains of nonsense-mediated decay factor UPF2
Source: Nucleic Acids Res. 2013 Nov 23;42(4):2673–86. doi: 10.1093/nar/gkt1197 (PMC3936715; doi:10.1093/nar/gkt1197)
Supplement: Supplementary Data [file supp_42_4_2673__index.html]

Structural and functional analysis of the three MIF4G domains of nonsense-mediated decay factor UPF2 — Structural and functional analysis of the three MIF4G domains of nonsense-mediated decay factor UPF2 — Supplementary Data 

# Structural and functional analysis of the three MIF4G domains of nonsense-mediated decay factor UPF2

## Supplementary Data

files

**Files in this Data Supplement:**

- Supplementary Data - pdf file
