# Supplementary material for: Structural and functional analysis of the three MIF4G domains of nonsense-mediated decay factor UPF2
Source: Nucleic Acids Res. 2013 Nov 23;42(4):2673–86. doi: 10.1093/nar/gkt1197 (PMC3936715; doi:10.1093/nar/gkt1197)
Supplement: Supplementary Data [file supp_gkt1197_nar-01980-v-2013-File009.pdf]

Structural and functional analysis of the three MIF4G domains of nonsense mediated decay factor UPF2

Supplementary Material

Marcello Clerici[1,2,4], Aurélien Deniaud[1,2,4], Volker Boehm[3], Niels H. Gehring[3], Christiane Schaffitzel[1,2,5] and Stephen Cusack[1,2,5]

[1] European Molecular Biology Laboratory, Grenoble Outstation, 6 rue Jules Horowitz, 38042 Grenoble Cedex 9, France

[2] Unit of Virus Host-Cell Interactions, Univ. Grenoble Alpes-EMBL-CNRS, UMI 3265, 6 rue Jules Horowitz, 38042 Grenoble Cedex 9, France

[3] University of Cologne, Institute for Genetics, Zuelpicher Str. 47a, 50674 Cologne, Germany

[4] These authors contributed equally.

[5] Corresponding authors.

Contents:

Supplementary text
Materials and methods
Supplementary references
Supplementary tables
Supplementary figures S1-S12

Supplementary text

Structural conservation of MIF4G domains.

A global pair-wise structure comparison (DaliLite, http://www.ebi.ac.uk/Tools/dalilite/) of UPF2 MIF4G-1 and MIF4G-2 domains with other MIF4G domain structures of eIF4G, CBP80, UPF2 MIF4G-3 and MIF4Gdb (PDB accession codes 1HU3, 1H6K, 1UW4 and 2I2O respectively) (Bae, 2010; Kadlec, 2004; Marcotrigiano, 2001; Mazza, 2001) indicates that core MIF4G domains share a similar overall fold with RMSDs between 2.0 and 4.0 Å, despite low sequence identity of between 10 and 23% (Supplementary Table 3, Supplementary Figure S4B). UPF2 MIF4G-1 has the highest structural similarity to eIF4G MIF4G domain (Cα RMSD 2.9 Å, residues 752-986, 13% identity), whereas MIF4G-2 is most similar to MIF4G-3 (Cα RMSD 2.5 Å, residues 768-983, 16% identity) (Supplementary Table 3). The identity in primary sequence does not correlate with the similarity in the structure, in agreement with the observation that structure-based alignments do not show any strictly conserved residues in MIF4G domains with the exception of the FIGEL motif (see below). Rather, they contain a pattern of hydrophobic residues important for interhelical packing (Kadlec, 2004). The structural comparison also shows a conservation of the position of the helices, with the exception of the N-terminal four-helix bundle of MIF4Gdb. The helix length is also generally conserved, with the exception of UPF2 MIF4G-1 helices h9 and h10 which are elongated (Supplementary Figure S4B). In contrast, the loops connecting the helices vary considerably. UPF2 MIF4G-1 and CBP80 contain an additional helix inserted between helices h8 and h9 and helices h9 and h10 respectively. All MIF4G domains share a conserved 'FIGEL' sequence motif on helix h6, with the hydrophobic residues being involved in interhelical packing and the glutamic residue pointing to the exterior (Kadlec, 2004; Letunic, 2002). In MIF4G-1, the glycine residue is mutated into an alanine (268-FIAEL-272), while the canonical motif is present in MIF4G-2 (669-FIGEL-673). In UPF2

MIF4G-1 and MIF4G-2, the exposed glutamic residue of the FIGEL motif is involved in the binding of the capping helix hA: in MIF4G-1, Glu271 establishes a salt bridge with Arg141 of helix hA (Figure 2A); in MIF4G-2, Glu672 interacts with helix hA forming a hydrogen bond with the hydroxyl group of a conserved tyrosine, Tyr468 (Figure 3A). In the light of these observations, we re-examined the context of the first, canonical, MIF4G domain of human CBP80. The ten helices of this domain are followed by a long proline-rich linker (residues 245-308), containing three short helices, which completely encircles the domain (Figure 1 of (Mazza, 2001) and Supplementary Figure S12A) making many specific contacts with it (Supplementary Figure S12B). Some regions of this linker make analogous interactions to that observed in the UPF2 MIF4G-2 structure. In particular Asp133 of the 'FIGEL' motif (129-FLSDL-134 in human CPB80) makes a hydrogen bond with Tyr253 of the linker (Supplementary Figure S12B). Thus, it seems possible to distinguish two categories of MIF4G domains. On one hand those such as the MIF4G domains of eIF4G, MIF4Gdb and UPF2 MIF4G-3 which are stable as 'naked' canonical MIF4G domains. On the other hand, those where the interhelical packing is stabilised by peptide extensions to the core domain, perhaps to limit flexibility of the helical bundle, such as the first two MIF4G domains of UPF2 and that of CBP80. Very recently, the structure of the MIF4G domain of Not1 has been published as part of the yeast Ccr4-Not complex (Basquin, 2012; Fabian, 2013; Petit, 2012). This shows another example of stabilisation of the core helical fold by N- and C-terminal extensions that wrap around the helices.

Materials and Methods

Protein expression, purification and crystallisation

DNA fragments encoding his-tagged UPF2(121-1031), UPF2(121-486), UPF2(455-757) and UPF2(455-1054) were cloned into the pProExHTb expression vector (Invitrogen). Plasmids were transformed into *E. coli* BL21Star(DE3), and the cells grown overnight at 20°C after induction with 1 mM IPTG. The proteins were purified by immobilised Ni^{2+} affinity chromatography. After His-tag removal using TEV protease (leaving a Gly-Ala-Met-Gly extension at the N-terminus), the UPF2 constructs were loaded a second time onto a Ni^{2+} column. The last purification step included size exclusion chromatography (Superdex 200) in 20 mM Tris pH 7, 150 mM NaCl and 2 mM DTT followed by concentration of the proteins. Crystallisation trials were performed with a Cartesian robot which makes 100nl+100nl drops, and positive hits conditions were refined by the hanging-drop vapour diffusion technique. UPF2-MIF4G-1 (121-486) crystals were obtained in 100 mM bicine pH 9, 100 mM NaCl, 11% PEG 6000 and at a protein concentration of 16 mg/ml; UPF2 MIF4G-2 (455-757) crystals were obtained in 100 mM MES pH 6.0, 19% PEG 3350 and at a protein concentration of 8 mg/ml; UPF2 MIF4G-2/MIF4G-3 (455-1054) crystals were obtained in 100 mM HEPES pH 7.0, 100 mM NH_4SO_4, 200 mM $NaKHPO_4$ pH 7, 840 mM sodium malonate, 1% w/v PEG MME 2K and at a protein concentration of 10 mg/ml. Expression of seleno-methionine labelled MIF4G-1, MIF4G-2 and MIF4G-2/MIF4G-3 was carried out in *E. coli* BL21Star(DE3) growing in M9 minimal medium. Thirty minutes before induction (1 mM IPTG) the cells were supplemented with a cocktail of aminoacids containing seleno-methionine (final concentration 60 mg/l). The labelled proteins were purified and crystallised in the same conditions as the native ones.

Crystallographic data collection and structure determination

Crystallographic statistics are given in Supplementary Table I. All data collection was performed at 100K at the European Synchrotron Radiation Facility with crystals being briefly soaked in a solution containing mother liquor and 20% glycerol as cryoprotectant and snap-frozen into liquid nitrogen. The data were integrated with XDS (Kabsch, 2010) and analysed with CCP4i. Molecular replacement was performed with PHASER (McCoy, 2007), model building with COOT (Emsley, 2010) and refinement with REFMAC5 (Murshudov, 1997; Vagin, 2004). As molecular replacement with previously known MIF4G domains did not work, the structures of both MIF4G-1 and MIF4G-2 were solved *de novo* by selenomethionine (SeMet) SAD, using SHELXD (Sheldrick, 2008) to find sites and SHARP (De la Fortelle, 1997) for refinement and phasing. For the MIF4G-1 structure, the SeMet data was the best quality and therefore used for refinement. For the low resolution combined MIF4G-2/MIF4G-3 domain structure, molecular replacement using PHASER (McCoy, 2007) using the individual domains, gave a unambiguous unique solution with log likelihood gain (LLG) of 220. This was confirmed by correspondence of predicted methionine positions with anomalous difference peaks using data from MIF4G-2/MIF4G-3 crystals grown with SeMet. Due to the low resolution, no further refinement was performed.

Generation of the quasi-atomic UPF-EJC model

Starting from the EM reconstruction of the UPF-EJC complex (EMD-2048) and the corresponding quasi-atomic model (Melero, 2012), we replaced the UPF2 MIF4G domains and the UPF3b RRM domain by our crystal structure of MIF4G-1 domain and by the MIG4G-2/3/UPF3b RRM complex. We used Chimera (Pettersen, 2004) to obtain the best correlation coefficient for the placement of the domains into the density. To avoid a clash with MIF4G-1 and MIF4G-2 (both are larger than the MIF4G homology model used in the original atomic model), the EJC had to be moved by 14 Å away from UPF2. Similarly, the

UPF1 CH-domain was moved by 5 Å away from UPF2 MIF4G-3 (Supplementary Figure S7). The figures were generated in PyMOL (DeLano Scientific). In the rendering of the cryo-EM map, the density cutoff was set for the display of the envelope to represent ~130% of the a priori estimated volume.

***In vivo* NMD assays**

The β-globin 4MS2 plasmid construct and the transfection control (wt300+e3) for the tethering assay were described previously (Gehring, 2005; Gehring, 2003). HeLa cells were grown in DMEM and transfected by calcium phosphate precipitation in 6-well plates with 0.8 µg of MS2-UPF2 fusion constructs, 0.5 µg of the control plasmid, 2 µg of the 4MS2 reporter plasmid, and 0.5 µg of a GFP expression plasmid. Total RNA was extracted with TRI Reagent (Sigma) and analyzed by northern blotting as described (Gehring, 2005; Gehring, 2003). Signals were quantified using a Typhoon Trio (GE Healthcare) and percentages ± standard deviations were calculated from three independent experiments.

For NMD complementation experiments HeLa cells were grown in 6 cm plates and transiently transfected with 100 pmol siRNA (UPF2 target sequence: 5'-CACGTTGTGGATGGAGTGTTA-3'; Luc target sequence: 5'-CGTACGCGGAATACTTCGATT-3') using Lipofectamine RNAiMAX (Life Technologies). On the next day, cells were split 1:2 into 10 cm plates and 24 h later transfected with 200 pmol siRNA. The next day, cells were seeded into 6-well plates and transfected by calcium phosphate precipitation with 0.5 µg of a GFP expression plasmid, 0.8 µg of expression plasmid for FLAG or siRNA insensitive (targeting sequence 5'-CACGTTGTGGATGGAGTGTTA-3' replaced by 5'-CATGTGGTTGACGGCGTCCTG-3') UPF2, SS166/167AA, S1046A and Δcoiled coil, 0.4 µg plasmid for ΔN/M1 and ΔN/M1M2,

0.6 µg for ΔM1 and ΔM2, 1.5 µg control plasmid (LacZ-HBB) and 2 µg plasmid encoding the reporter mRNA (TPI-HBB) (Steckelberg, 2012), harbouring a stop codon at position 48.

For UPF2 localisation HeLa cells were transfected with 600 ng of pCI-mVenus-UPF2 plasmids. Cells were plated on coverslips 24 hours post transfection. 48 hours after transfection cells were fixed with 3.7% formaldehyde. Images were recorded on a FV1000 confocal microscope (Olympus).

Immunoprecipitation, immunoblotting and antibodies

SDS-polyacrylamide gel electrophoresis and immunoblot analysis was performed using protein samples derived either from TRI Reagent extractions (Figure 5D) or from lysates (RIPA buffer supplemented with protease inhibitor) of parallel transfections (Figure 5B). For coimmunoprecipitation UPF2 mutants were expressed as FLAG-tagged proteins in HeLa cells. Fusion proteins were immunoprecipitated with FLAG-beads (Sigma-Aldrich) and co-purified proteins analysed by immunoblotting. The antibodies against tubulin (T6074) and FLAG (F7425) were from Sigma, the antibody against V5 (18870) was from QED Bioscience, the antibody against GFP (ab290) was from Abcam and the antibodies against UPF1 and UPF2 were kindly provided by Jens Lykke-Andersen. UPF3B antiserum was raised in rabbits by Eurogentech against a C-terminal fragment of UPF3B (300-483) and affinity purified.

SMG1 purification

SMG1 was expressed and purified as described in (Izumi, 2010) with some modifications. Lysed cells in SMG1 buffer (20 mM Hepes pH 7.5, 100 mM NaCl, 1 mM $MgCl_2$, 0.5 mM DTT, 0.05% tween-20, 5% glycerol) supplemented with protease and phosphatase inhibitors (Roche) were ultracentrifuged at 100 000x g for 30 minutes. The

supernatant was mixed with streptavidin beads and incubated for 2 hours at 4 °C with mixing. After washing with SMG1 buffer containing 100 mM NaCl and then 500 mM NaCl, SMG1 was eluted with SMG1 buffer with 1.5 mM biotin. Subsequently, SMG1 was concentrated and applied on a Superose-6 column equilibrated with SMG1 buffer. The fractions corresponding to monomeric SMG1 were pooled, concentrated, snap-frozen and stored at -80 °C.

SMG1 kinase assay

0.2 µg of SMG1 were mixed with 1 µg of either UPF1-FL, UPF2 (121-486), UPF2 (455-757) or UPF2 (761-1054) in SMG1 buffer containing 2 mM DTT, 5 mM $MnCl_2$ and 5 mM ATP. The mixture was incubated 1 h at room temperature. Proteins were separated on 12 % SDS-PAGE gels first stained with Pro-Q Diamond Staining (Life Technologies) and then with Coomassie staining. The Pro-Q stained gel was revealed with a Typhoon scanner using 532 nm and 580 nm as excitation and emission wavelength, respectively.

Surface Plasmon Resonance

SPR experiments were performed on a BIAcore 3000 using SA sensor chips (GE-Healthcare). One flow-cell was not functionalized to be used as a background control. The second flow-cell was functionalized with purified SMG1 to a density of about 2000 RU. The running buffer was 20 mM Hepes pH 7.5, 100 mM NaCl, 1 mM $MgCl_2$, 0.5 mM DTT, 0.05% Tween-20. UPF2/UPF3b solutions were injected at 25 µl/min during 3 or 4 min followed by a 10 min dissociation phase. The surfaces were regenerated by 1 minute injection of 0.5 M and 1 M NaCl solutions. The data were analyzed with the Biacore evaluation software by subtracting both the control flow cell and the buffer injection curve. The apparent equilibrium dissociation constants ($K_{D\text{-app}}$) were determined using RU values 10 seconds before the end of

the association phase for all curves (RU_{max}). These RU_{max} were plotted as a function of protein concentration and fitted assuming a one binding site model.

Phosphosites identification by mass spectrometry

500 µg of UPF2 (761-1054) in SMG1 buffer containing 2 mM DTT, 5 mM $MnCl_2$ and 5 mM ATP were mixed with or without 10 µg of SMG1. The mixtures were incubated 5 h at room temperature. UPF2 and SMG1 were then separated using a Superose-6 column equilibrated with SMG1 buffer containing 500 mM NaCl. The peak corresponding to UPF2 was collected and concentrated to 100 µl. Samples were then diluted with 50mM NH_4HCO_3 to obtain 20 µL of 0.5 µg/µl of protein , which was reduced with DTT (50 mM, 2 µL) for 30 min at 56 °C and alkylated with iodacetamide (110 mM, 2µL) for 20 min at room temperature in the dark. Incubation with trypsin (0.1 µg enzyme/10 µg protein) was carried out overnight at 37 °C. Digestion was stopped by adding 4 µl of 10% trifluoracetic acid. Prior to analysis by liquid chromatography - mass spectrometry (LC-MS/MS), the peptides were diluted to 0.1 µg/µl with 0.1% formic acid. Peptides were separated using the nanoAcquity UPLC system (Waters) fitted with a trapping (nanoAcquity Symmetry C18, 5µm, 180 µm x 20 mm) and an analytical column (nanoAcquity BEH C18, 1.7µm, 75µm x 200mm). The outlet of the analytical column was coupled directly to an LTQ Orbitrap Velos (Thermo Fisher Scientific) using the Proxeon nanospray source. The mass spectrometric raw data was processed using MaxQuant (version 1.1.1.36) (Cox, 2008).

Supplementary references

Bae, E., E. Bitto, C.A. Bingman, J.G. McCoy, G.E. Wesenberg and G.N. Phillips, Jr. (2010). Crystal structure of an eIF4G-like protein from Danio rerio. *Proteins* **78**(7): 1803-6.

Basquin, J., V.V. Roudko, M. Rode, C. Basquin, B. Seraphin and E. Conti (2012). Architecture of the nuclease module of the yeast Ccr4-not complex: the Not1-Caf1-Ccr4 interaction. *Mol Cell* **48**(2): 207-18.

Cox, J. and M. Mann (2008). MaxQuant enables high peptide identification rates, individualized p.p.b.-range mass accuracies and proteome-wide protein quantification. *Nat Biotechnol* **26**(12): 1367-72.

De la Fortelle, E., J.J. Irwin and G. Bricogne (1997). SHARP: A Maximum-Likelihood Heavy-Atom Parameter Refinement and Phasing Program for the MIR and MAD Methods. *Crystallographic computing*.

Emsley, P., B. Lohkamp, W.G. Scott and K. Cowtan (2010). Features and development of Coot. *Acta Crystallogr D Biol Crystallogr* **66**(Pt 4): 486-501.

Fabian, M.R., F. Frank, C. Rouya, N. Siddiqui, W.S. Lai, A. Karetnikov, P.J. Blackshear, B. Nagar and N. Sonenberg (2013). Structural basis for the recruitment of the human CCR4-NOT deadenylase complex by tristetraprolin. *Nat Struct Mol Biol* **20**(6): 735-9.

Gehring, N.H., J.B. Kunz, G. Neu-Yilik, S. Breit, M.H. Viegas, M.W. Hentze and A.E. Kulozik (2005). Exon-junction complex components specify distinct routes of nonsense-mediated mRNA decay with differential cofactor requirements. *Mol Cell* **20**(1): 65-75.

Gehring, N.H., G. Neu-Yilik, T. Schell, M.W. Hentze and A.E. Kulozik (2003). Y14 and hUpf3b form an NMD-activating complex. *Mol Cell* **11**(4): 939-49.

Izumi, N., A. Yamashita, A. Iwamatsu, R. Kurata, H. Nakamura, B. Saari, H. Hirano, P. Anderson and S. Ohno (2010). AAA+ proteins RUVBL1 and RUVBL2 coordinate PIKK activity and function in nonsense-mediated mRNA decay. *Sci Signal* **3**(116): ra27.

Kabsch, W. (2010). Xds. *Acta Crystallogr D Biol Crystallogr* **66**(Pt 2): 125-32.

Kadlec, J., E. Izaurralde and S. Cusack (2004). The structural basis for the interaction between nonsense-mediated mRNA decay factors UPF2 and UPF3. *Nat Struct Mol Biol* **11**(4): 330-7.

Letunic, I., L. Goodstadt, N.J. Dickens, T. Doerks, J. Schultz, R. Mott, F. Ciccarelli, R.R. Copley, C.P. Ponting and P. Bork (2002). Recent improvements to the SMART domain-based sequence annotation resource. *Nucleic Acids Res* **30**(1): 242-4.

Marcotrigiano, J., I.B. Lomakin, N. Sonenberg, T.V. Pestova, C.U. Hellen and S.K. Burley (2001). A conserved HEAT domain within eIF4G directs assembly of the translation initiation machinery. *Mol Cell* **7**(1): 193-203.

Mazza, C., M. Ohno, A. Segref, I.W. Mattaj and S. Cusack (2001). Crystal structure of the human nuclear cap binding complex. *Mol Cell* **8**(2): 383-96.

McCoy, A.J. (2007). Phaser crystallographic software. *J. Appl. Crystallogr.* **40**: 658-674.

Melero, R., G. Buchwald, R. Castano, M. Raabe, D. Gil, M. Lazaro, H. Urlaub, E. Conti and O. Llorca (2012). The cryo-EM structure of the UPF-EJC complex shows UPF1 poised toward the RNA 3' end. *Nat Struct Mol Biol* **19**(5): 498-505, S1-2.

Murshudov, G.N., A.A. Vagin and E.J. Dodson (1997). Refinement of macromolecular structures by the maximum-likelihood method. *Acta Crystallogr D Biol Crystallogr* **53**(Pt 3): 240-55.

Petit, A.P., L. Wohlbold, P. Bawankar, E. Huntzinger, S. Schmidt, E. Izaurralde and O. Weichenrieder (2012). The structural basis for the interaction between the CAF1 nuclease and the NOT1 scaffold of the human CCR4-NOT deadenylase complex. *Nucleic Acids Res* **40**(21): 11058-72.

Pettersen, E.F., T.D. Goddard, C.C. Huang, G.S. Couch, D.M. Greenblatt, E.C. Meng and T.E. Ferrin (2004). UCSF Chimera--a visualization system for exploratory research and analysis. *J Comput Chem* **25**(13): 1605-12.

Sheldrick, G.M. (2008). A short history of SHELX. *Acta Crystallogr A* **64**(Pt 1): 112-22.

Steckelberg, A.L., V. Boehm, A.M. Gromadzka and N.H. Gehring (2012). CWC22 connects pre-mRNA splicing and exon junction complex assembly. *Cell Rep* **2**(3): 454-61.

Vagin, A.A., R.A. Steiner, A.A. Lebedev, L. Potterton, S. McNicholas, F. Long and G.N. Murshudov (2004). REFMAC5 dictionary: organization of prior chemical knowledge and guidelines for its use. *Acta Crystallogr D Biol Crystallogr* **60**(Pt 12 Pt 1): 2184-95.

	UPF2 MIF4G-1 (121-486) SeMet data	UPF2 MIF4G-2 (455-757) Native data	UPF2 MIF4G-2 (455-757) SeMet data	UPF2 MIF4G-2/3 (455-1054) Native data	UPF2 MIF4G-2/3 (455-1054) SeMet data
**Data collection (date)**	07/07/2007	15/02/2009	07/03/2009	10/04/2008	07/10/2008
Beamline (ESRF)	BM14	ID23-2	ID14-4	ID14-4	ID14-4
Wavelength (Å)	0.9788	0.8726	0.9795	0.9395	0.9795
Space group	$P6_5$	$P2_12_12_1$	$P2_12_12_1$	$I2_12_12_1$	$I2_12_12_1$
Cell dimensions a, b, c (Å)	101.1, 101.1, 158.9	40.9, 56.6, 142.2	41.3, 56.7, 145.8	92.5, 132.1, 216.6	95.4, 132.4, 218.5
α, β, γ (°)	90, 90, 120	90, 90, 90	90, 90, 90	90, 90, 90	90, 90, 90
Resolution (Å) *	50-2.60 (2.70-2.60)	50-2.35 (2.40-2.35)	50-3.10 (3.10-3.20)	50-5.4 (5.8-5.4)	50-6.0 (6.0-6.3)
R_{merge}*	0.089 (0.63)	0.066 (0.81)	0.104 (0.848)	0.068 (0.68)	0.058 (0.466)
$I / \sigma I$*	10.9 (1.7)	13.8 (1.9)	10.5 (1.7)	10.7 (2.0)	9.2 (1.7)
Completeness (%)*	99.1 (99.3)	99.7 (99.4)	99.9 (100)	95.9 (97.0)	97.4 (98.0)
Redundancy*	2.86 (2.37)	3.95 (4.09)	3.98 (4.00)	3.58 (3.65)	1.94 (1.95)
**Refinement**					
Resolution (Å) *	50-2.6 (2.67-2.60)	50-2.35 (2.41-2.35)			
No. reflections work/free	26788/1428	13638/723			
R_{work}*	0.205 (0.327)	0.206 (0.286)			
R_{free}*	0.251 (0.420)	0.251 (0.348)			
No. atoms	5115	1990			
Protein atoms	5061 (2 copies)	1958 (1 copy)			
Water molecules	54	32			
Average B-factors (Å^2)	55.2	53.4			
R.m.s. deviations					
Bond lengths (Å)	0.007	0.012			
Bond angles (°)	1.059	1.462			
Ramachandran Plot**					
Favoured (%)	96.1	94.8			
Allowed (%)	99.2	100			

* Values in parentheses are for highest-resolution shell. ** Molprobity http://molprobity.biochem.duke.edu

Supplementary Table 1. Data collection and refinement statistics for UPF2 domains.

Identified peptide	Phosphorylated residue	Score
LCNSLEESIR	S992	188.85
MVESAVIFR	S886	179.75
DSMTEGENIEEDEEEEEGGAETEEQSGNESEVNE	T1042 or S1046 or S1050	136.66

Supplementary Table 2. Identification of the SMG-1 *in vitro* phosphorylation sites of UPF2 by mass spectrometry (MS). Based on MS, only one site is phosphorylated in the third peptide.

	eIF4G	MIF4Gdb	MIF4G-1	MIF4G-2	MIF4G-3	CBP80
**eIF4G (1HU3, 744-986)**	-					
**MIF4Gdb (2I2O, 7-217)**	2.9	-				
**MIF4G-1 (121-429)**	2.9	3.3	-			
**MIF4G-2 (458-757)**	3.8	3.4	3.5	-		
**MIF4G-3 (1UW4, 768-983)**	3.6	3.7	3.7	2.5	-	
**CBP80 (1H6K, 26-244)**	3.5	3.2	4.0	2.5	2.0	-

Supplementary Table 3. RMSD (Å) of Cα position for the superposition of MIF4G domains of published structures and UPF2 MIF4G-1 and 2. PDB accession codes and domain boundaries used for superposition are indicated in brackets. The structures were superposed with DaliLite (http://www.ebi.ac.uk/Tools/structure/dalilite).

A

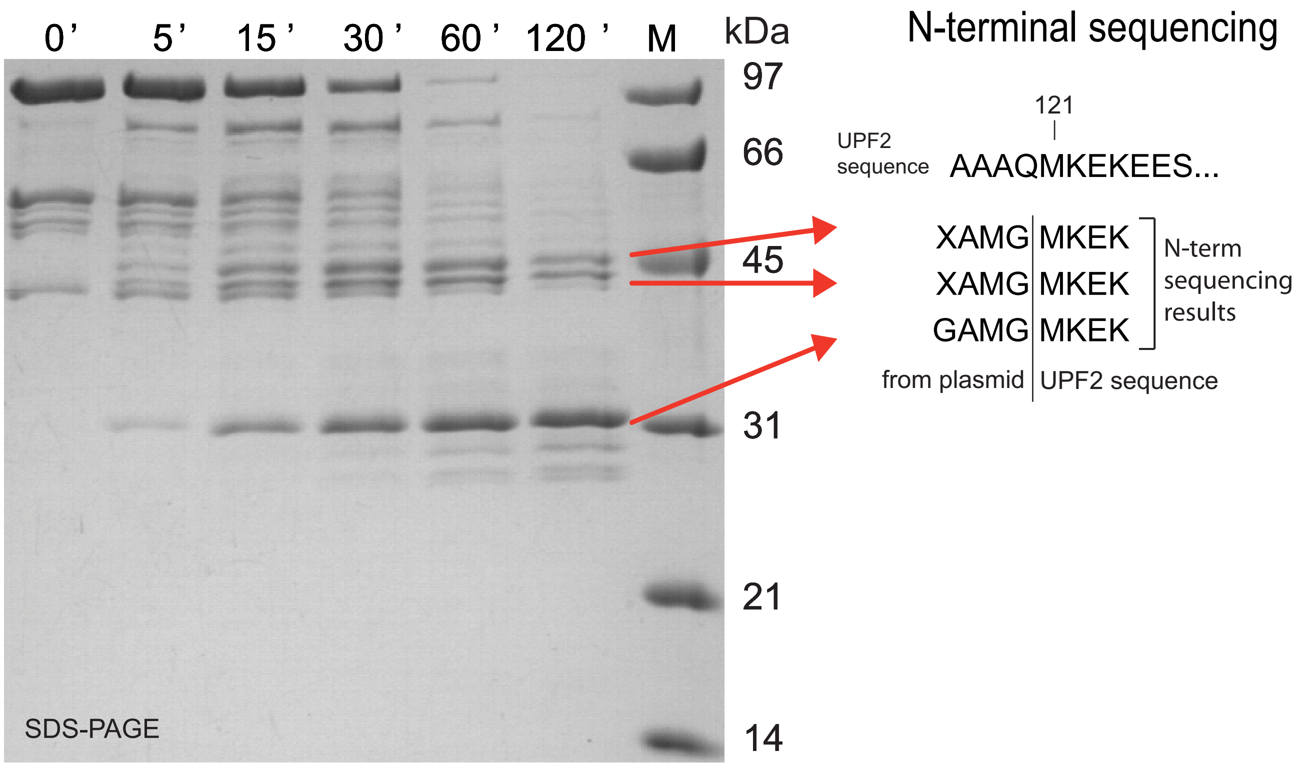

B

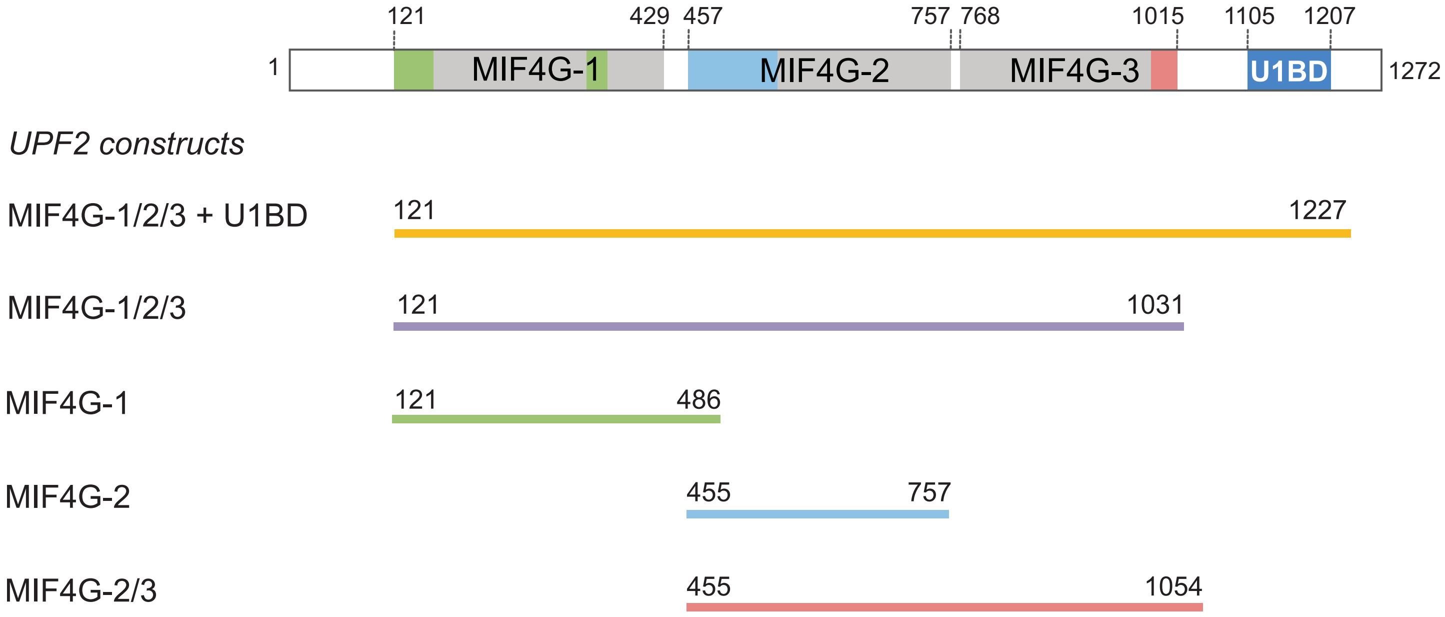

Supplementary Figure S1. A. UPF2(121-1031) limited proteolysis time course and results of N-terminal sequencing on stable bands. UPF2(121-1031) was mixed with trypsin in 1000:1 weight ratio and the reaction stopped at different time points and loaded on SDS-PAGE gel. Stable bands were excised from the gel and analysed by N-terminal sequencing. The N-terminal four residues GAMG are left on the protein from the plasmid used for expression after TEV cleavage. **B**. Schematic representation of UPF2 (rectangle) as in figure 1A. UPF2 constructs used in this study are shown as lines.

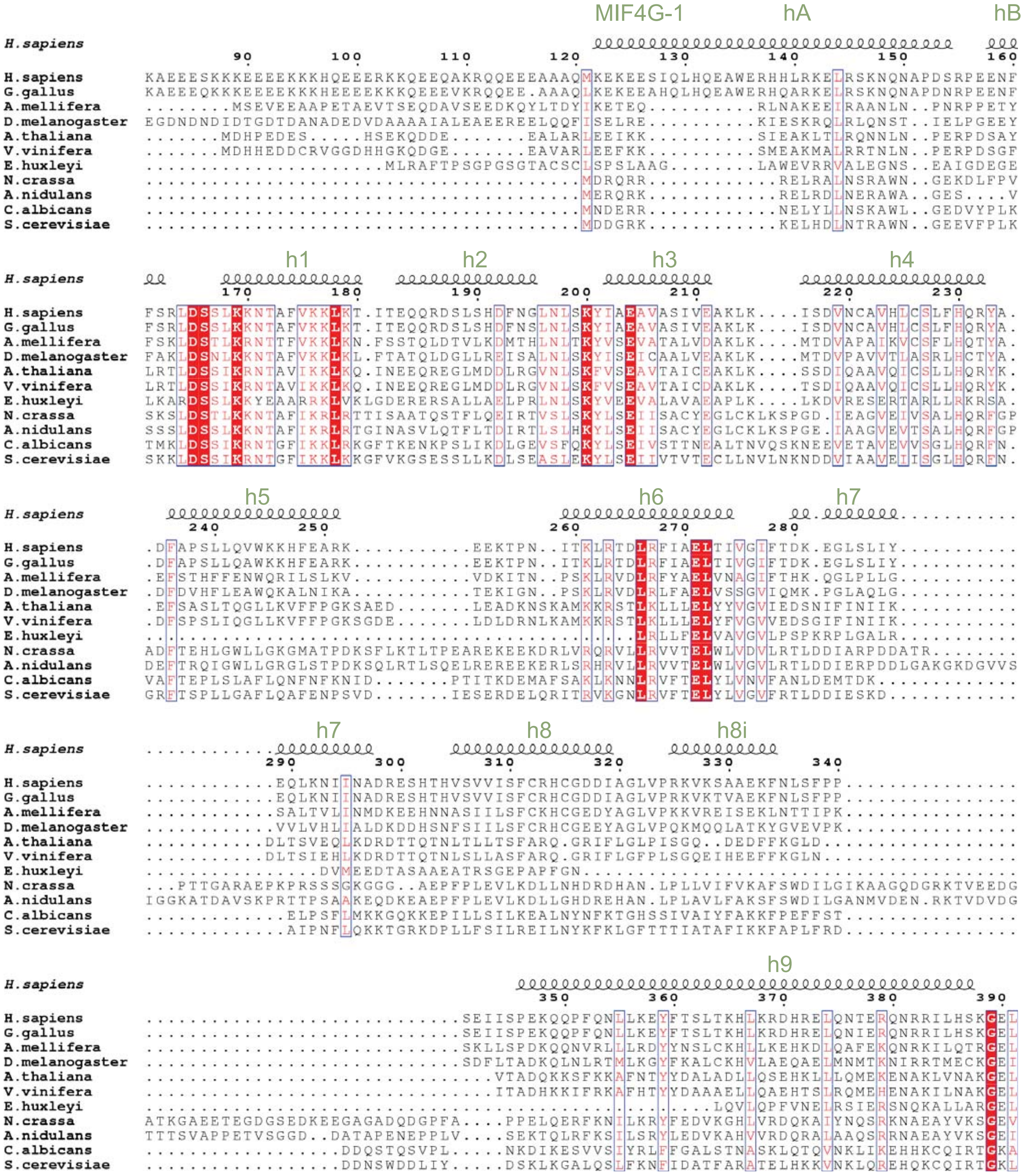

Supplementary Figure S2. (continues in the next page)

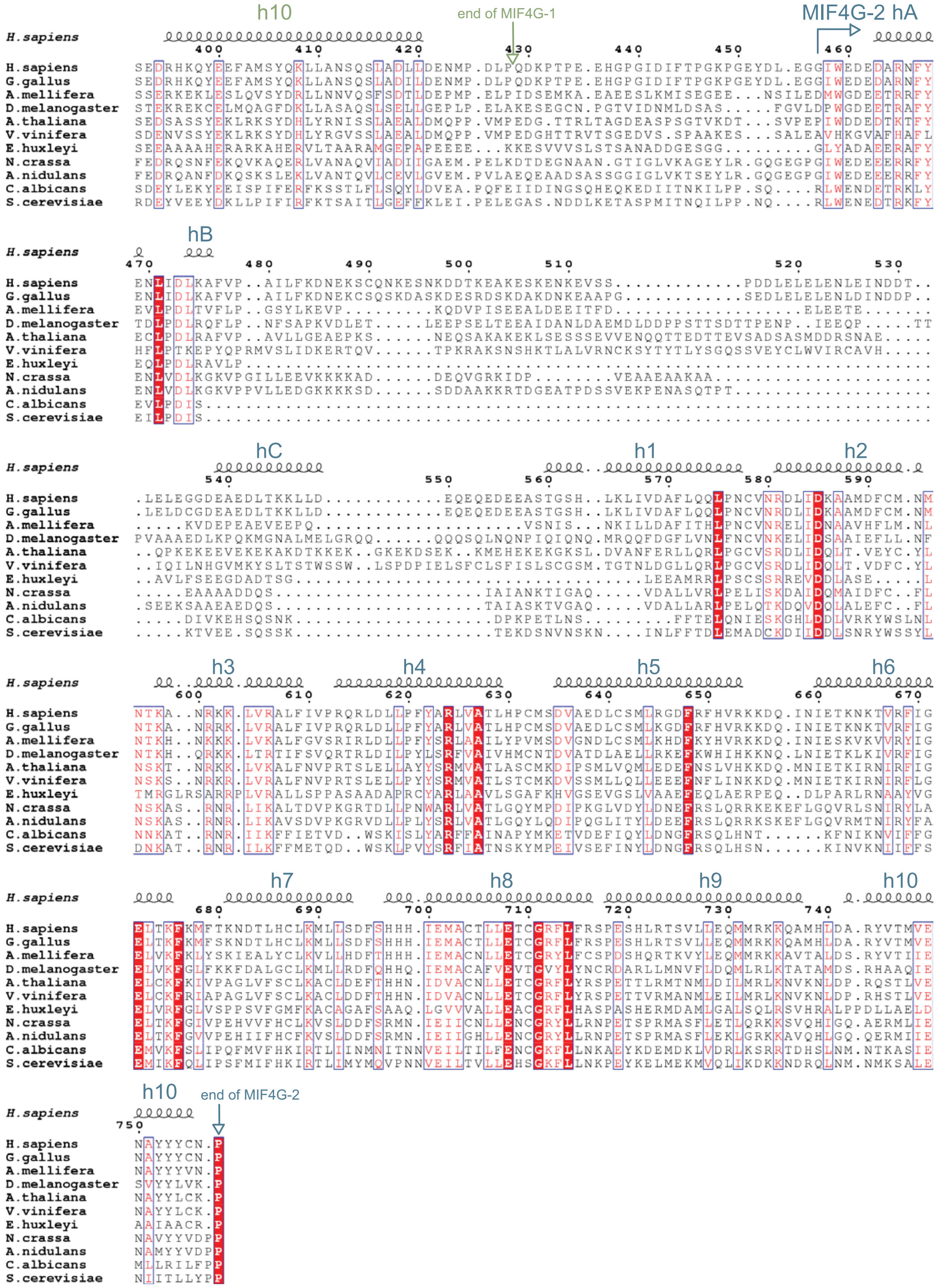

Supplementary Figure S2. Sequence alignment of UPF2 MIF4G domains 1 and 2 of representative UPF2 proteins from yeast to human (Accession numbers. Hs: AAG60689; Gg: XP_004937542; Am: XP_003249451; Dm: NP_572434; At: NP_199512; Vv: XP_002275646; Eh: EOD19187; Nc: XP_961757.2; An: XP_664299.1 ; Ca: XP_720987.1; Sc: NP_011944.2). Residues with similarity >85% are displayed in red. The secondary structure (all alpha-helices) of human UPF2 MIF4G domains 1 and 2 are indicated in green and blue respectively.

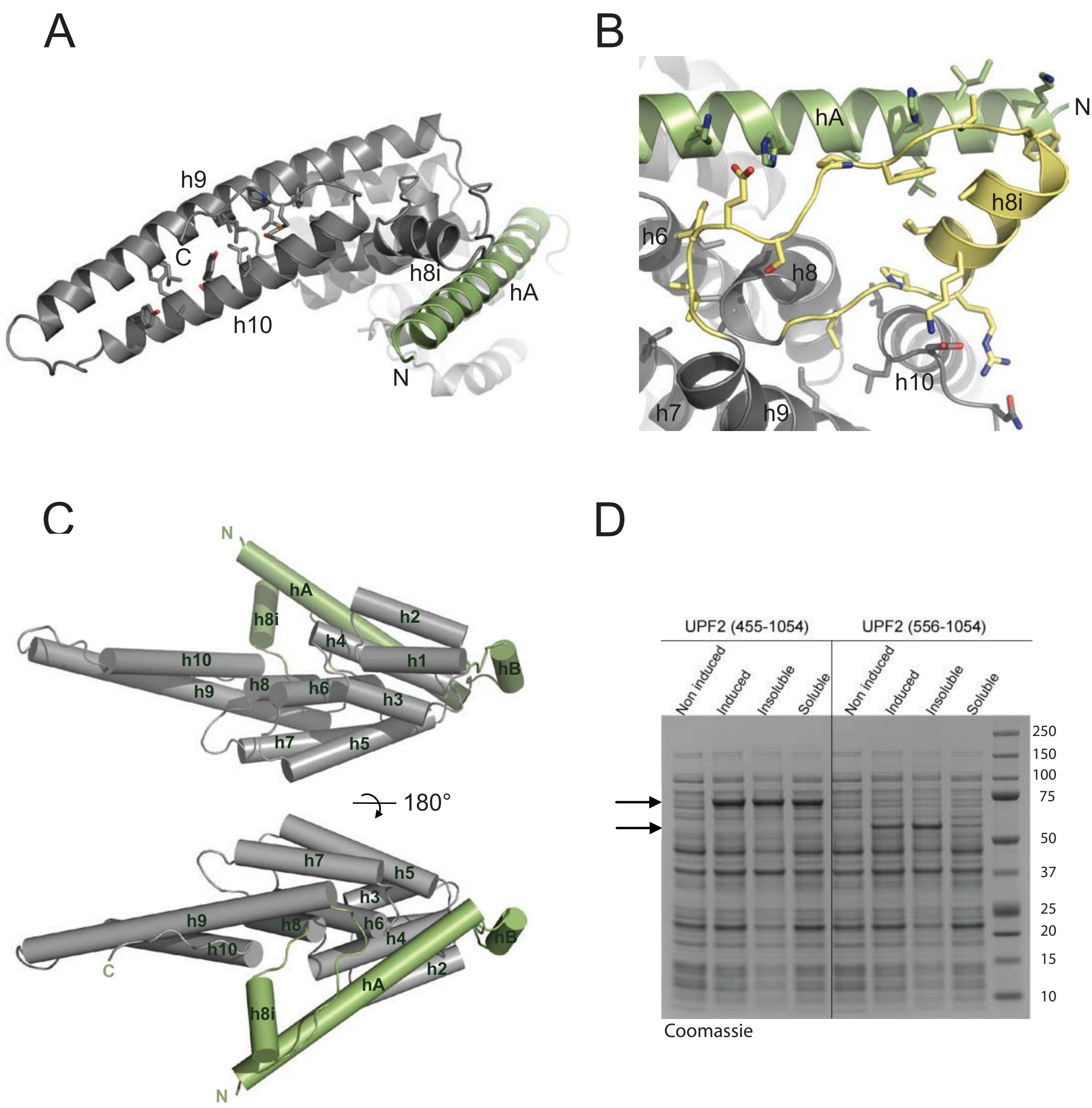

Supplementary Figure S3. A. Helices h9 and h10 of UPF2 MIF4G-1 form an elongated coiled-coil structure protruding away from the domain. Hydrophobic residues involved in helices h9 and h10 packing are depicted as sticks. **B**. Detailed view of the interaction between MIF4G-1 helix h8i with its N- and C-terminal loops and helices h6, h8 and h10. Residues involved in the packing are depicted as sticks. **C**. MIF4G-1 domain represented in the same orientations as in figure 1C. **D**. Coomassie-stained SDS-PAGE of total lysate (non induced and induced), soluble and insoluble fractions of UPF2 MIF4G-2/3 constructs with MIF4G-2 helices hA-hB and disordered linker (constructs 455-1054) and without (556-1054) showing that In the absence of helices hA and hB the solubility of UPF2 MIF4G-2/3 is reduced.

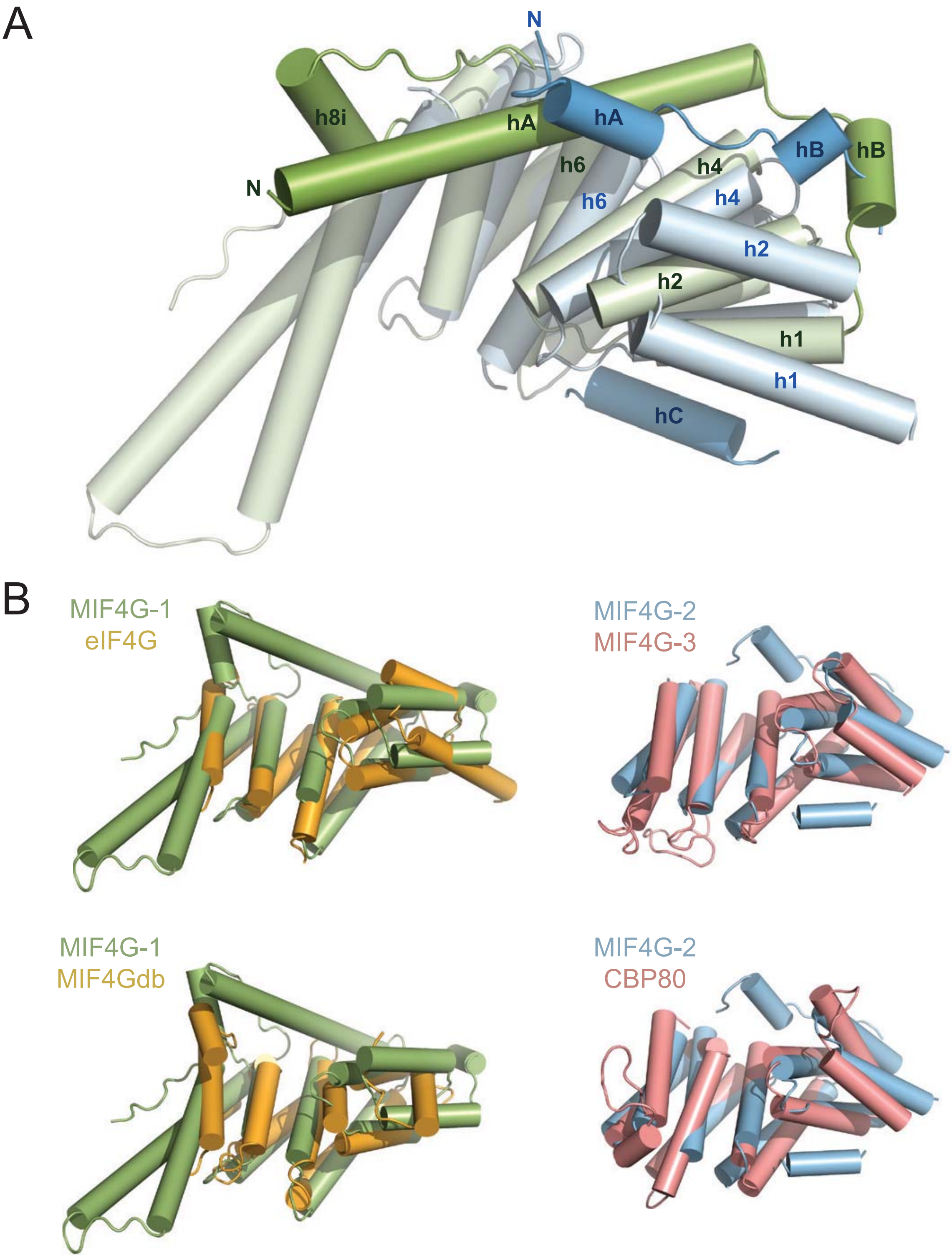

Supplementary Figure S4. A. Superposition of UPF2 MIF4G-1 (light green) and MIF4G-2 (light blue) showing the partial overlap of MIF4G-1 helix hA (green) with MIF4G-2 helix hA (blue). **B**. Superposition of UPF2 MIF4G-1 (green) to eIF4G and MIF4Gdb MIF4G domains (orange); superposition of UPF2 MIF4G-2 (light blue) to UPF2 MIF4G-3 and CBP80 MIF4G domain (pink). Helices are represented as cylinders.

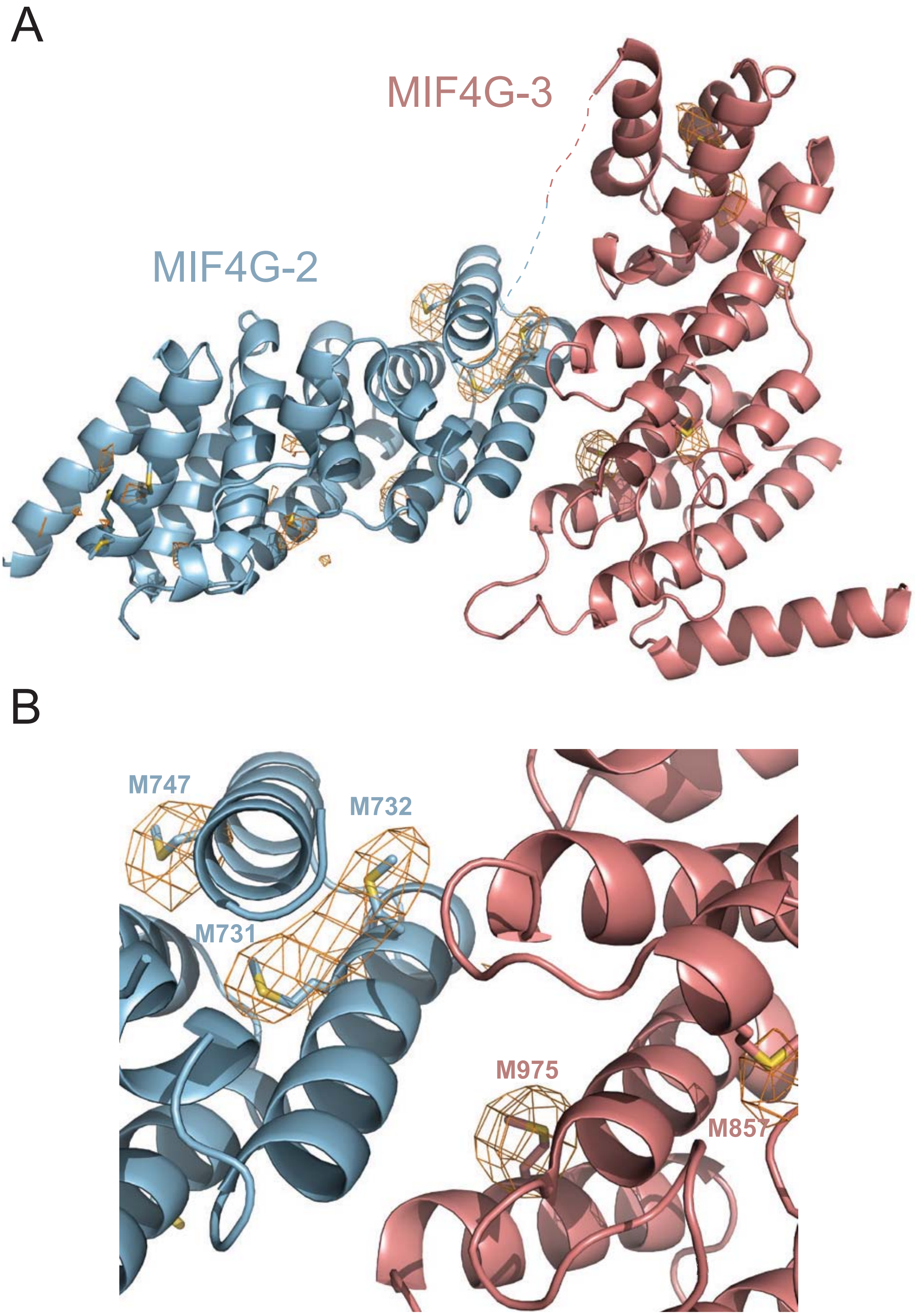

Supplementary Figure S5. A. Anomalous difference map (orange mesh) of seleno-methionine substituted UPF2 MIF4G-2/MIF4G-3 domains (contoured at 3.5 σ) indicating the position of the methionine residues (depicted as sticks). **B**. Detailed view of the anomalus difference map in the MIF4G-2/MIF4G-3 interaction region; methionine residues are labeled.

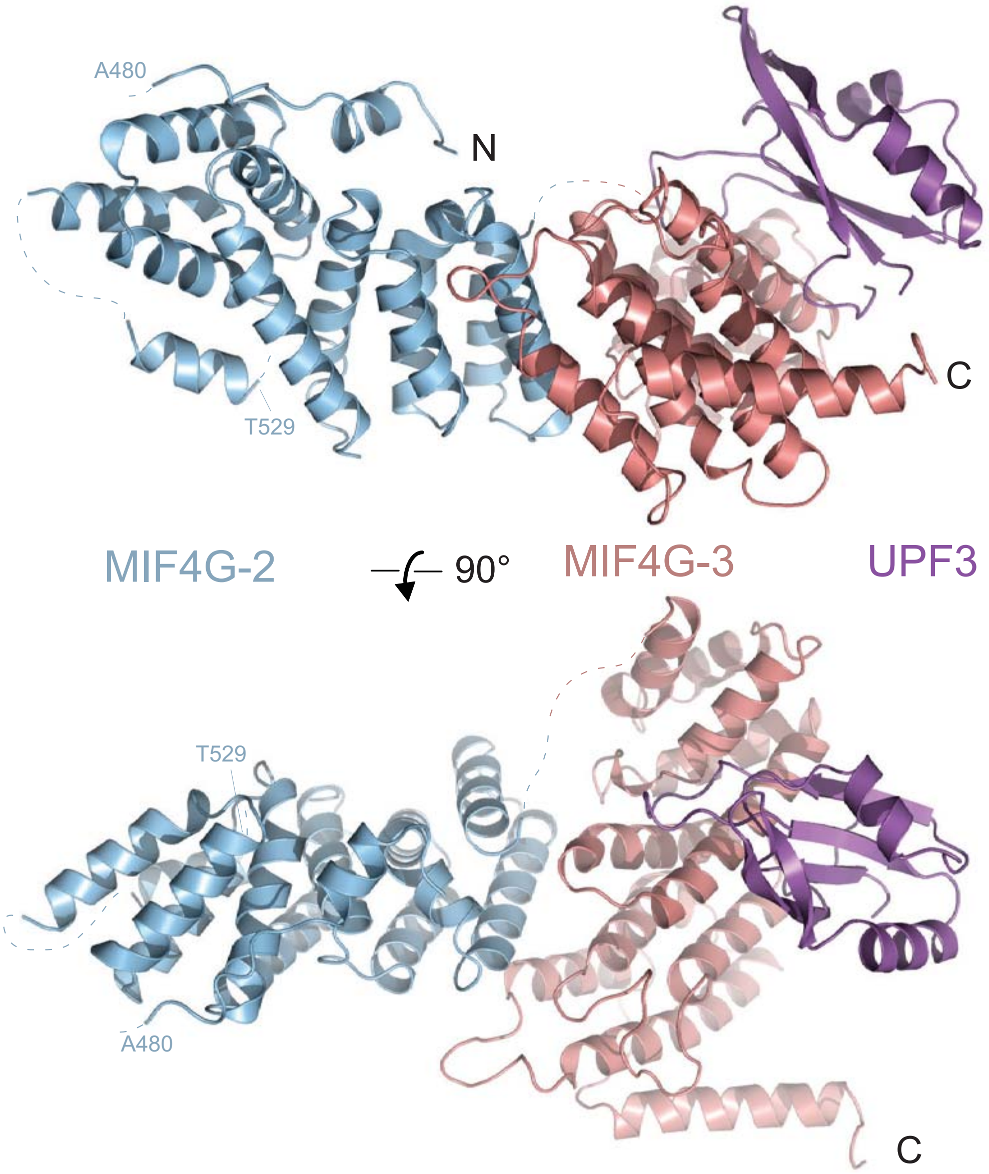

Supplementary Figure S6. Two views of the reconstructed UPF2 MIF4G-2/MIF4G-3 assembly in complex with UPF3 RRM domain. The complex was obtained by superposition of MIF4G-3 in the UPF2-UPF3 complex and in the MIF4G-2/MIF4G-3 assembly.

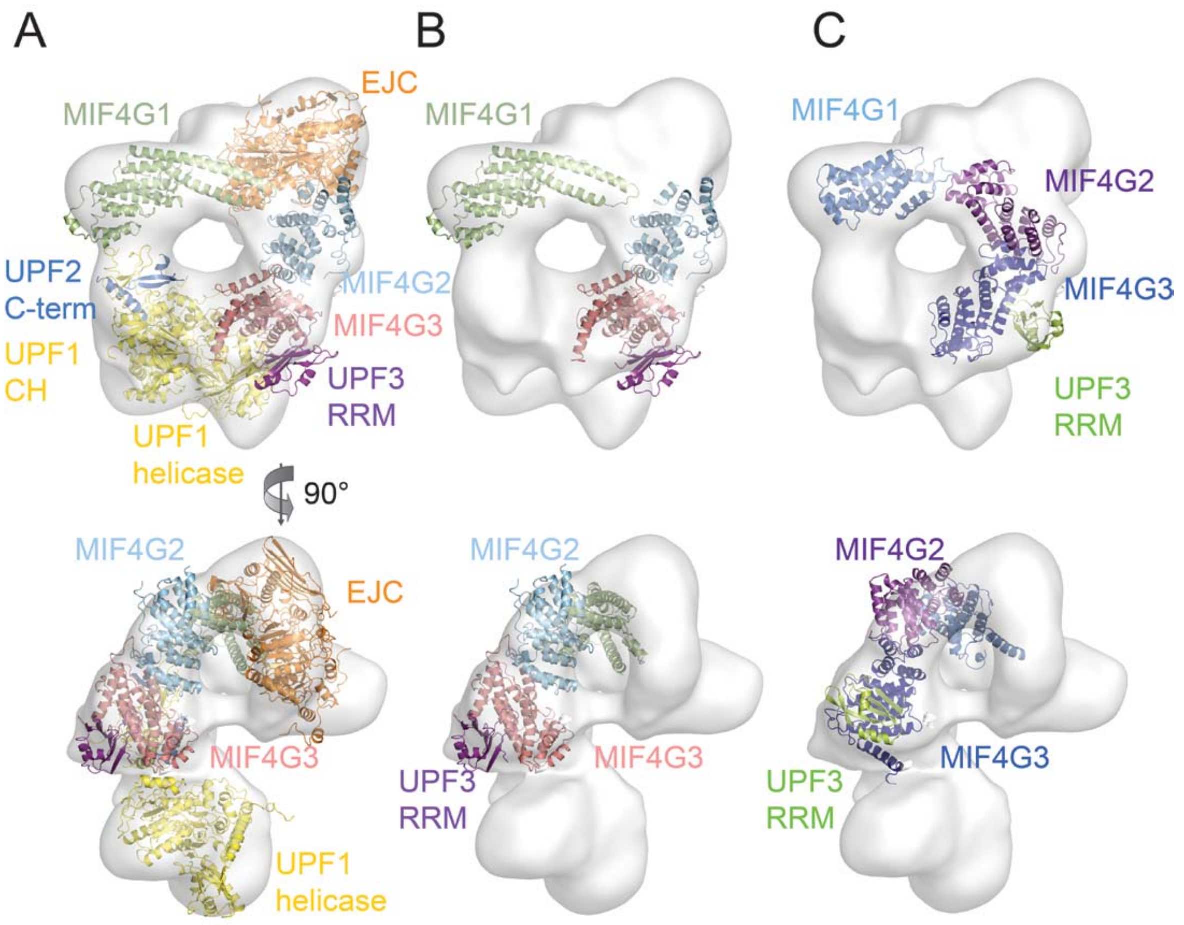

D

Proteins	Correlation Coefficient, published model	Correlation Coefficient, new model
UPF2 MIF4G-domain 1	0.51	0.59
UPF2 MIF4G-domain 2&3 — UPF3 RRM	0.45	0.47
UPF1 CH-domain — UPF2 C-terminus	0.54	0.70
UPF1 helicase	0.34	0.34
EJC — UPF3	0.53	0.58

Supplementary Figure S7 A. Fitting of the EJC-UPF3, UPF1 complex with the UPF2 C-terminus, UPF2 MIF4G-1 (this study) and MIF4G-2/MIF4G-3/UPF3 RRM (this study) crystal structures into the cryo-EM reconstruction of the UPF-EJC complex (EMD-2048, Melero et al., 2012). The view in the lower panels is rotated 90 degrees with respect to the upper panel. **B**. The same representation as in A showing only the MIF4G-1 and MIF4G-2/MIF4G-3/UPF3 RRM structures (this study) placed into the EM reconstruction. **C**. Original quasi-atomic model showing the MIF4G-1 homology model, the MIF4G-2 homology model and the MIF4G-3/UPF3 RRM crystal structure placed into the cryo-EM reconstruction (Melero et al., 2012). **D**. Table listing the correlation coefficients obtained by rigid body fitting of UPF domains and the EJC for the published quasi-atomic model and for the new model. The overall quality of the fitting is very similar for the two quasi-atomic models. The correlation coefficients were determined using Chimera.

A

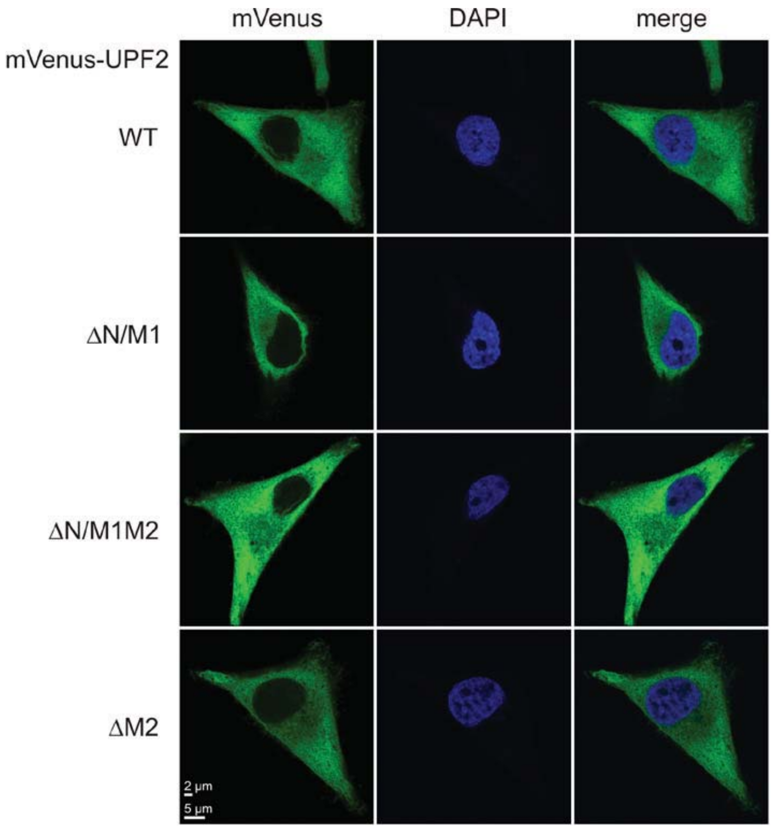

B

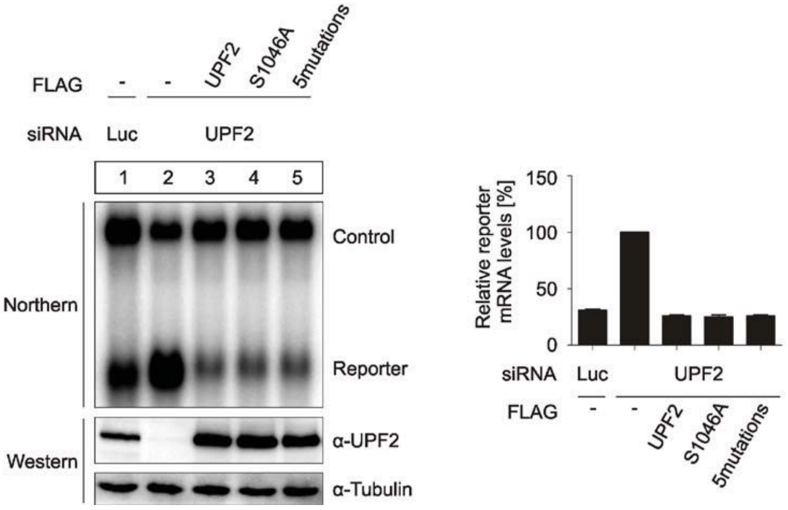

Supplementary Figure S8. A. Subcellular localisation of UPF2 mutants. HeLa cells were transfected with plasmids expressing the indicated mVenus-UPF2-constructs and transferred to coverslips 24 hours afterwards. Cells were fixed 48 hours post-transfection and analysed by confocal microscopy. Images of representative cells from each transfection are shown. Scale bars = 2 and 5mm. All analysed UPF2 mutants localise to the cytoplasm. **B**. Complementation assay performed using UPF2 S1046A mutant and UPF2 carrying all five phosphorylation sites mutations (S886A, S992A, T1042A, S1046A and S1050A). The assay was performed as described in the legend of figure 5B.

A

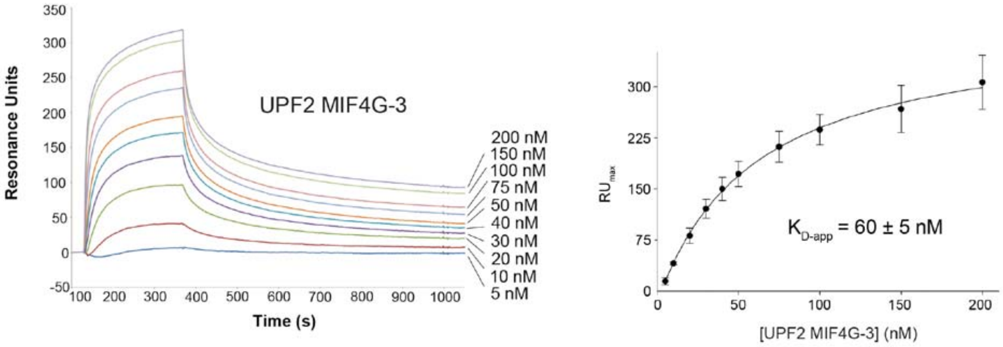

B

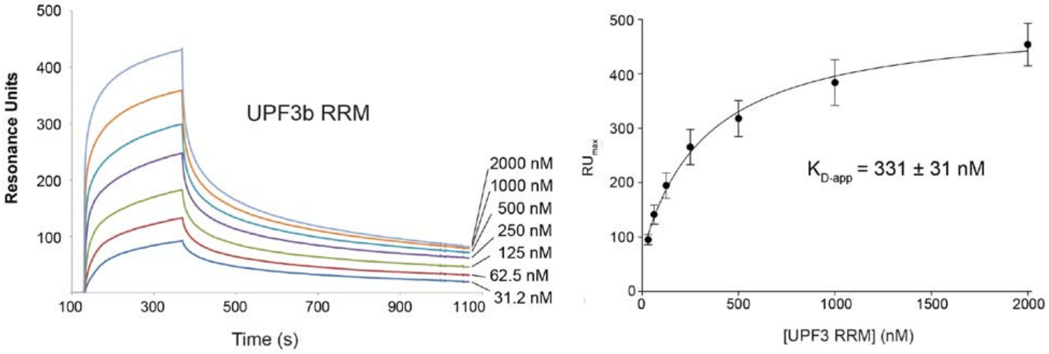

Supplementary Figure S9. Overlay of SPR sensograms of **(A)** UPF2 MIF4G-3 and **(B)** UPF3b RRM interacting with immobilized SMG1. UPF2 MIF4G-3 was injected at concentrations ranging from 5 nM to 200 nM. UPF3 RRM was injected at concentrations ranging from 31.2 nM to 2000 nM. The maximal resonance unit signal was plotted against the injected protein concentration (right panels); the data points result from three independent experiments. The apparent dissociation constants were determined assuming a 1:1 interaction.

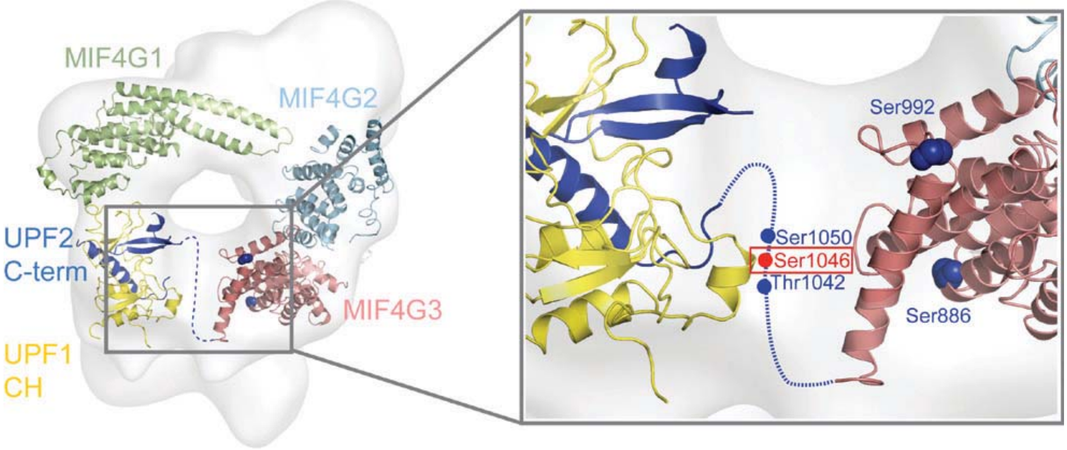

Supplementary Figure S10. Location of the SMG1 phosphorylation sites of UPF2 identified by in vitro phosphorylation and mass spectroscopy of UPF2. Phosphorylated serines are depicted as spheres in the quasi-atomic model of the UPF2 – UPF1 CH domain complex which is shown together with the UPF-EJC EM density (Melero et al., 2012). The main in vitro phosphorylation site of UPF2 (Ser1046) is highlighted in red; it is located on a flexible linker between the MIF4G domain 3 and the UPF2 C-terminal domain

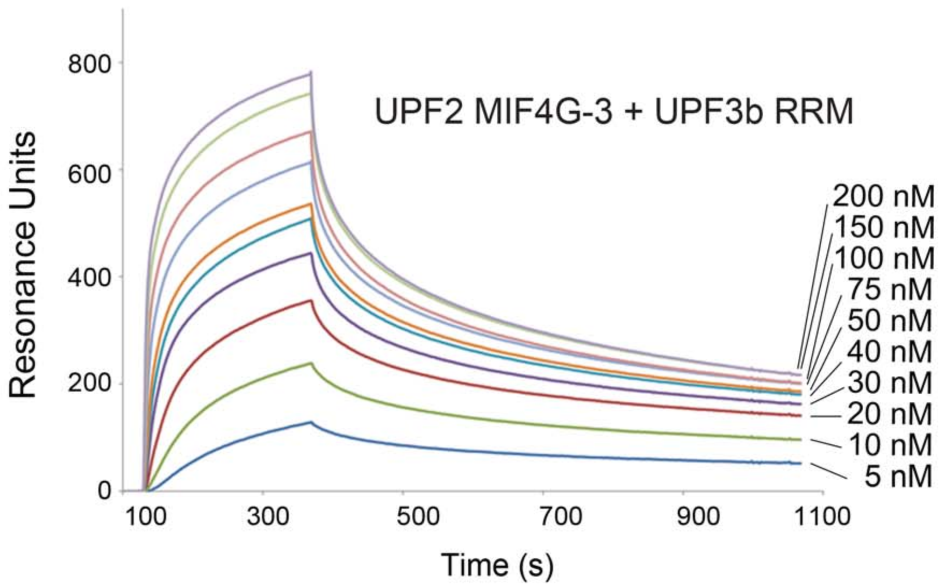

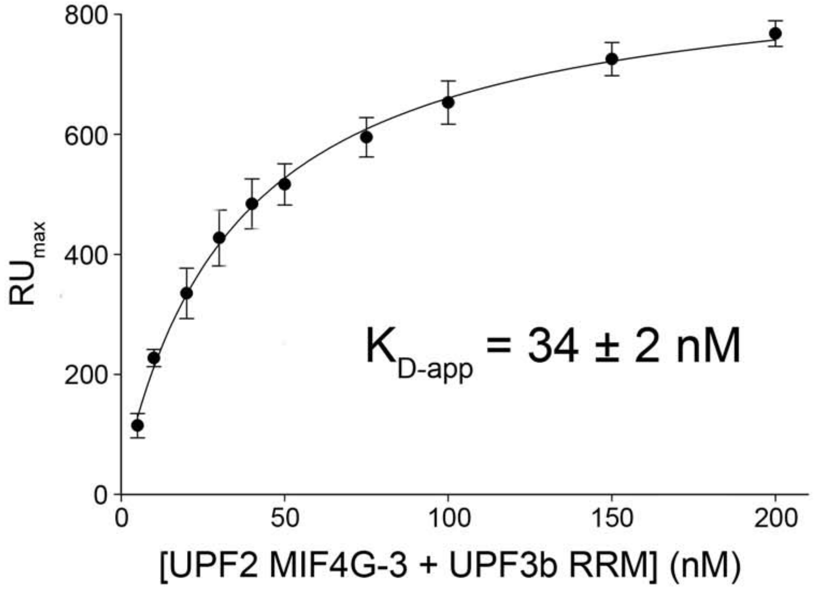

Supplementary Figure S11. Overlay of SPR sensograms of the preformed UPF2 MIF4G-3/UPF3b RRM complex interacting with immobilized SMG1. The UPF2/UPF3 complex was injected at concentrations ranging from 5 nM to 200 nM. The maximal resonance unit signal was plotted against the injected protein concentration; the data points result from three independent experiments. The apparent dissociation constant was determined assuming a 1:1 interaction.

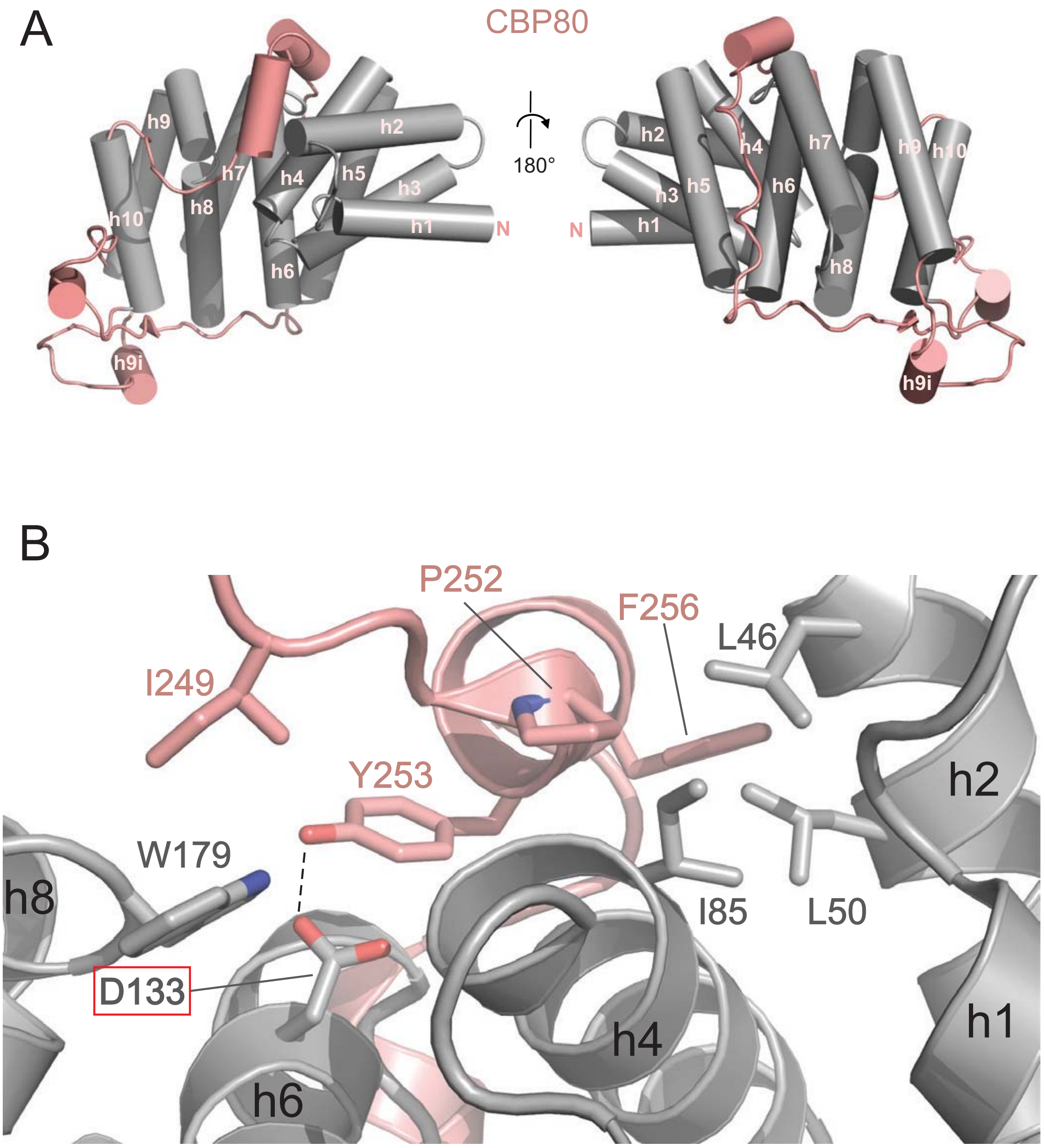

Supplementary Figure S12. A. Structure of human CBP80 MIF4G-1 domain. The ten helices (grey) of the MIF4G domain fold are numbered from one to ten. The extended C-terminal linker of the domain (which contains three helices) and the additional helix (h9i) inserted between h9 and h10 are depicted in pink. **B**. Helix annotation and colour code as in (A). Detailed view of one of the helices of the extended C-terminal linker which interacts tightly with helices h2, h4, h6 and h8 of the core domain. Amino acids involved in the interaction are shown in stick representation. Notably, aspartic acid (D133) of the conserved FIGEL signature motif (FLSDL in CBP80) of MIF4G domains in helix h6 interacts with tyrosine 253 of the C-terminal linker.
